# Overexpression of BIRC6 Is a Predictor of Prognosis for Colorectal Cancer

**DOI:** 10.1371/journal.pone.0125281

**Published:** 2015-05-01

**Authors:** Tingting Hu, Shuqiang Weng, Wenqing Tang, Ruyi Xue, She Chen, Guoxiang Cai, Yu Cai, Xizhong Shen, Si Zhang, Ling Dong

**Affiliations:** 1 Department of Gastroenterology and Hepatology, Shanghai Institute of Liver Disease, Zhongshan Hospital, Fudan University, Shanghai, China; 2 Department of Gastroenterology and Hepatology, the First Affiliated Hospital of Wenzhou Medical University, Wenzhou, China; 3 Department of Biochemistry and Molecular Biology, Shanghai Medical College, Fudan University, Shanghai, China; 4 Department of Colorectal Surgery, Fudan University Shanghai Cancer Center, Fudan University, Shanghai, China; 5 Department of Oncology, Shanghai Medical College, Fudan University, Shanghai, China; Baylor University Medical Center, UNITED STATES

## Abstract

**Background and Objective:**

Inhibitors of apoptosis proteins (IAPs) have been well investigated in human cancers, where they are frequently overexpressed and associated with poor prognosis. Here we explored the role of baculoviral IAP repeat containing 6 (BIRC6), a member of IAPs, in human colorectal cancer (CRC).

**Methods:**

We used Western blotting and immunohistochemistry to examine BIRC6 expression in 7 CRC cell lines and 126 CRC clinical samples. We determined the biological significance of BIRC6 in CRC cell lines by a lentivirus-mediated silencing method.

**Results:**

We reported that BIRC6 was overexpressed in CRC cell lines and clinical CRC tissues. BIRC6 overexpression was correlated with tumor size and invasion depth of CRC. BIRC6 overexpression is associated with worse overall survival (OS) (*P* = 0.001) and shorter disease-free survival (DFS) (*P* = 0.010). BIRC6 knockdown inhibited cell proliferation, arrested cell cycle at S phase, downregulated cyclin A2, B1, D1 and E1 levels, and sensitized CRC cells to chemotherapy *in vitro* and *in vivo*.

**Conclusions:**

Taken together, these data suggests that BIRC6 overexpression is a predictor of poor prognosis in colorectal cancer and BIRC6 could be a potential target of CRC therapy.

## Introduction

Colorectal cancer (CRC) is the third most frequently diagnosed malignancy and the fourth leading cause of cancer death worldwide[1]. Due to screening and removal of premalignant polyps, incidence rates have declined over the last 3 decades[2]. The use of new drugs such as oxaliplatin, bevacizumab, cetuximab and panitumumab allows patients with metastatic colorectal cancer survive longer[3,4,5,6]. Despite the progress in the diagnosis and treatment of CRC, the mortality from this disease remains high. Given the poor prognosis of CRC, novel prognostic markers and therapeutic strategies need to be developed to further improve the outcome of colorectal cancer.

The inhibitors of apoptosis protein (IAP) family has been demonstrated to be crucial in apoptosis resistance in a wide range of malignancy[7,8,9,10]. These proteins are distinguished by the presence of up to three copies of Baculoviral IAP Repeat (BIR) domain. The IAPs bind to and suppress a variety of pro-apoptotic factors, thereby effectively inhibit apoptosis in cancer cells[11]. Baculoviral inhibition of apoptosis protein repeat containing 6 (BIRC6), also known as Apollon or Bruce, is the largest member of the IAP family with a single BIR domain at its N-terminal and an active ubiquitin-conjugating (UBC) enzyme domain at its C-terminal[12]. In addition to its IAP activity, BIRC6 has the distinctive property of activity as a chimeric E2/E3 ubiquitin ligase in mammals[13]. With its UBC domain, BIRC6 promotes the degradation of Smac and inhibits the activity of caspase-9, which play key roles in the initiation of apoptosis[14]. While BIRC6 itself is regulated by ubiquitination and proteasomal degradation mediated by E2, UbcH5c and Nrdp1[15].

A large amount of evidence showed that BIRC6 was highly expressed in several types of cancer. It has been reported that brain cancer cells with high levels of BIRC6 were resistant to various anticancer drugs[12]. BIRC6 overexpression was associated with unfavorable prognosis in childhood *de novo* acute myeloid leukemia[16]. Moreover, it has been reported that p53 is a downstream effector of BIRC6[17,18]. These findings suggest that BIRC6 maybe a new therapeutic target for malignant tumor.

Qiu *et al*. [14] first proved that siRNAs targeting BIRC6 promoted apoptotic cell death. Subsequently, several studies confirmed that silencing BIRC6 caused elevated apoptosis[17,18,19]. In addition, preliminary investigation demonstrated that BIRC6 expression was more abundant in CRC tissues than in non-neoplastic tissues using cDNA microarrays[20]. Similar result was obtained in the comparative proteomics study of colon cancer stem cells[21]. However, there have been no reports of its prognostic relevance based on clinical data. In the present study, we explored the prognostic significance and biologic features of BIRC6 in colorectal cancer. Our data showed a strong correlation between the BIRC6 overexpression and the unfavorable clinical features. In addition, BIRC6 upregulation in CRC predicted poor prognosis of patients. Furthermore, We found that BIRC6 knockdown inhibited CRC cell proliferation, arrested CRC cell cycle at S phase, downregulated cyclin A2, B1, D1 and E1 levels, and sensitized CRC cells to chemotherapy.

## Materials and Methods

### Patients, tissue specimens and follow-up

The present study was predominantly conducted by a retrospective design. CRC samples (n = 126) were obtained from patients who received surgery at the Zhongshan Hospital, Fudan University between January 2008 and August 2008. None of them received radiotherapy. Among 126 patients, 42 Patients receieved oxaliplatin-based chemotherapy (FOLFOX4; oxaliplatin, 5-fluorouracil and leucovorin) after curative resection. The follow-up information of all participants was updated every 3 months by telephone. All patients were monitored prospectively by serum CEA, ultrasound examination, endoscope, computed tomography every 3–6 months. The diagnosis of recurrence was based on the imaging method and biopsy (if possible). Patients were followed until death or April 1, 2013, with a mean postoperative follow-up duration of 59 months. Overall survival (OS) was defined as the time from the date of surgery to death by any cause, patients alive being censored at the last follow-up. Disease-free survival (DFS) was defined as the time elapsed from surgery to the first occurrence of any of the following events: colorectal cancer distant metastasis; recurrence of colorectal cancer; development of second non-colorectal malignancy excluding basal cell carcinomas of the skin and carcinoma in situ of the cervix; or death from any cause without documentation of a cancer-related event. Patients with TNM stage IV tumors were excluded when analyzing DFS. Thirty paired fresh resection CRC tissues and the paired adjacent non-cancerous tissues were collected for Western blotting. The study was approved by the Research Ethics Committee of Zhongshan Hospital. All patients provided written consent forms for this study.

### Microsatellite instability (MSI) analysis and KRAS mutation analysis

MSI analysis was examined using three mononucleotide repeat markers: BAT25, BAT26, and CAT25 as described previously[22]. KRAS mutation analysis was performed by PCR amplification of genomic DNA using the following primers: sense 5’-AAGGCCTGCTGAAAATGACTG-3’ and antisense 5’-AGAATGGTCCTGCACCAGTAA-3’.

### Immunohistochemistry and evaluation

Paraffin-embedded sections of normal and tumor tissues were deparaffinized in xylene and rehydrated in a decreasing ethanol series diluted in distilled water. Following antigen retrieval with a 10 mM citrate buffer, CRC sections were incubated overnight at 4°C with the primary anti-BIRC6 polyclonal antibody (Abcam, Cambridge, MA). Following 30 min incubation with secondary antibody against HRP-conjugated-rabbit Ig, sections were developed in 3, 3’-diaminobenzidine solution under microscopic observation and counterstained with hematoxylin.

The sections were observed under a light microscopy, for a histological review, to examine tumor microheterogeneity in antigen distribution. Five randomized microscopic views of 400-fold magnification of each section were observed and scored. Both the intensity of immunohistochemical staining (0, negative; 1, weak; 2, intermediate; 3, strong) and the percentage of positive cells (0, 0% positive cells; 1, 1–10% positive cells; 2, 11–50% positive cells; 3, >50% positive cells) were evaluated. The final score of each sample was obtained by multiplying the scores of staining intensity and percentage of positive cells. Samples were classified as negative when the final scores were 0 to 3 and positive when 4 to 9 [23]. The BIRC6 staining was scored independently by two pathologists blinded to the clinical characteristics of the patients. The intra-observer reproducibility was tested by obtaining the widely used statistical κ-scores that grade the strength of agreement to six categories [poor (κ-score, <0.00), slight (0.00–0.20), fair (0.21–0.40), moderate (0.41–0.60), substantial (0.61–0.80) and almost perfect (0.81–1.00)][24].

### Cell culture

LoVo, SW620, DLD-1, HT-29, HCT116, SW480 and SW1116 were purchased from the Type Culture Collection of Chinese Academy of Sciences (Shanghai, China), human colon healthy cell line NCM460 were purchased from INCELL corporation. The SW620, HT-29, SW480 and HCT116 cells were routinely maintained in Dulbecco’s modified Eagle’s medium supplemented with 10% fetal bovine serum (FBS) (HyClone, Logan, UT). LoVo, DLD-1 and SW1116 were maintained in RPMI 1640 medium plus 10% fetal bovine serum. NCM460 were grown in M3:10 medium. Cells were maintained at 37°C in a humidified incubator under 5% CO_2_.

### Establishment of stable BIRC6-knockdown clones

Lentiviral transduction was used to establish BIRC6 knockdown stable clones. A panel of shRNA (short hairpin RNA) lentiviral vectors differed in BIRC6-targeting sequences (TRCN0000041-57~61) and pLKO.1-puro empty vector were purchased from Sigma Aldrich (St Louis, MO, USA). Cells were transfected at a MOI of 1 for 24h, screened by puromycin. The inhibition efficiency was identified by Western blotting.

### Western blotting

Colorectum tissue or harvested cells were homogenized in SDS lysis buffer (40 mM Tris pH 7.4, 150 mM NaCl, 1 mM EDTA, 1% SDS, 1 mM aprotinin, 1 mM PMSF and 10 mg/ml leupeptin) and then centrifuged for 15 min at 4°C. Total protein lysates extracted from samples were quantitated with BCA Protein Assay Kit (Pierce, Rockford, IL, USA). An aliquot of protein was boiled with protein loading buffer for 5 min, and was loaded on SDS polyacrylamide gel. Equal amounts of protein were separated by 8% SDS-PAGE and transferred to polyvinylidene difluoride membranes (Millipore, Billerica, MA, USA) using a mini trans-blot apparatus (Bio-Rad Laboratories, Hercules, CA, USA). Membranes were blocked with PBS-0.05% Tween 20 containing 5% nonfat dry milk for 1 h, followed by incubation with antibody against BIRC6 or glyceraldehyde-3-phosphate dehydrogenase antibody at 4°C overnight. The membrane was then incubated with secondary horseradish peroxidase-conjugated goat anti-rabbit or anti-mouse antibody (Jackson Immune Research Laboratories Inc, West Grove, PA). Blots were developed using an enhanced chemiluminescence kit (Tiangen, China). Each experiment was repeated at least three times.

### Cell proliferation assay

Cell proliferation was measured using the Cell Counting Kit-8 (CCK-8) (Dojindo Co., Kumamoto, Japan) according to the instructions of the supplier. Cells were incubated with CCK-8 for 1 hour with 3 multiples and proliferation rate was assessed by measuring the absorbance at 450 nm with the Universal Micro-plate Reader. Each experiment was repeated three times[25].

### Colony formation assay

Anchorage-independent growth ability was measured using soft agar colony formation assay and plate colony formation assay respectively. For soft agar colony formation, control or stable BIRC6-knockdown cells (1 × 10^3^ cells/well) were resuspended in DMEM or RPMI 1640 containing 0.3% noble agarose (Takara Shuzo Co., Tokyo, Japan) in six-well plates. The suspension was laid over DMEM or RPMI 1640 containing 0.6% noble agarose and further covered with 0.5 ml DMEM or RPMI 1640. The plates were then incubated in a 5% CO_2_ incubator at 37°C for 14 days, with replenishment of medium every other day. Colonies were imaged using Nikon ECLIPSE TE300 and macroscopically visible colonies in 3 randomly chosen fields per well were counted for quantification. For plate colony formation assay, cells from different treated groups were seeded into six-well plates (800 cells/well) for 2 weeks. Colonies were stained with 0.1% crystal violet in 20% methanol and colonies containing more than 50 cells were counted. Plating efficiency was calculated as: plating efficiency = (colony number/plating cell number) × 100%, in triplicate.

### Cell cycle and apoptosis assay

The effect of BIRC6 knockdown on the cell cycle was analyzed by flow cytometry. Cells were trypsinized and fixed with ice-cold 70% ethanol at 4°C overnight. For DNA content analysis, cells were incubated with 50 μg/mL propidium iodide (Sigma, St. Louis, MO) in the presence of 100 μg/mL RNase and 0.2% Triton X-100 for 30 minutes at 37°C. DNA content was determined in the BD FACSAria^II^. The distribution of cells in each phase of the cell cycle was calculated using the Flowjo Program. Each experiment was performed in triplicate.

For apoptosis analysis by flow cytometry, cells were treated with 5-FU, CDDP, oxaliplatin or CPT-11 (All from Sigma, St. Louis, MO)[26]. Drug-induced apoptosis was analyzed using Annexin V assay kit (BD Biosciences, San Jose, CA) according to the instructions of the manufacturer.

### Tumor xenograft models

Twenty male Balb/c nude mice (4 weeks of age, 12–14 g) were purchased from Experimental Animals Center of Shanghai Institute of Life Science (Shanghai, China) and were raised under specific pathogen-free conditions. All surgery was performed under anesthesia with sodium pentobarbital. DLD-1 cells (stable BIRC6 knockdown 59 and the control clones, 10^6^) in 0.1 ml of PBS were injected subcutaneously into the right flank of each mouse. Mice were sacrificed at 4 weeks post-injection; tumors were excised and weighed. Tumor volume was calculated by the formula: 0.5 × L × W^2^ (L = length of tumor; W = width of tumor). Animal experiments were performed according to the criteria outlined in the Guide for the Care and Use of Laboratory Animals, prepared by the National Academy of Sciences and published by the National Institutes of Health, and also approved by the ethics committee of Fudan University.

### Statistical analysis

Statistical analysis was performed using IBM SPSS statistical software (version 17.0). Categorical variables were compared by χ^2^ test or Continuity Correction or Fisher’s exact tests as appropriate. Continuous variables were compared using Wilcoxon ranksum test and independent two sample *t*-test. Univariate analysis were performed to identify the factors that affect survival of CRC. Multivariate analysis were done using the Cox multivariate proportional hazard regression model. Survival curves were estimated using the Kaplan-Meier method (the log-rank test). All data were presented as mean ± standard error of the mean (SEM) from three independent experiments and *P*-values were determined from 2-sided tests. Statistical significance was displayed as *, *P* < 0.05 or **, *P* < 0.01.

## Results

### Enhanced BIRC6 expression in CRC cells lines and clinic CRC tissues

**Fig 1:**
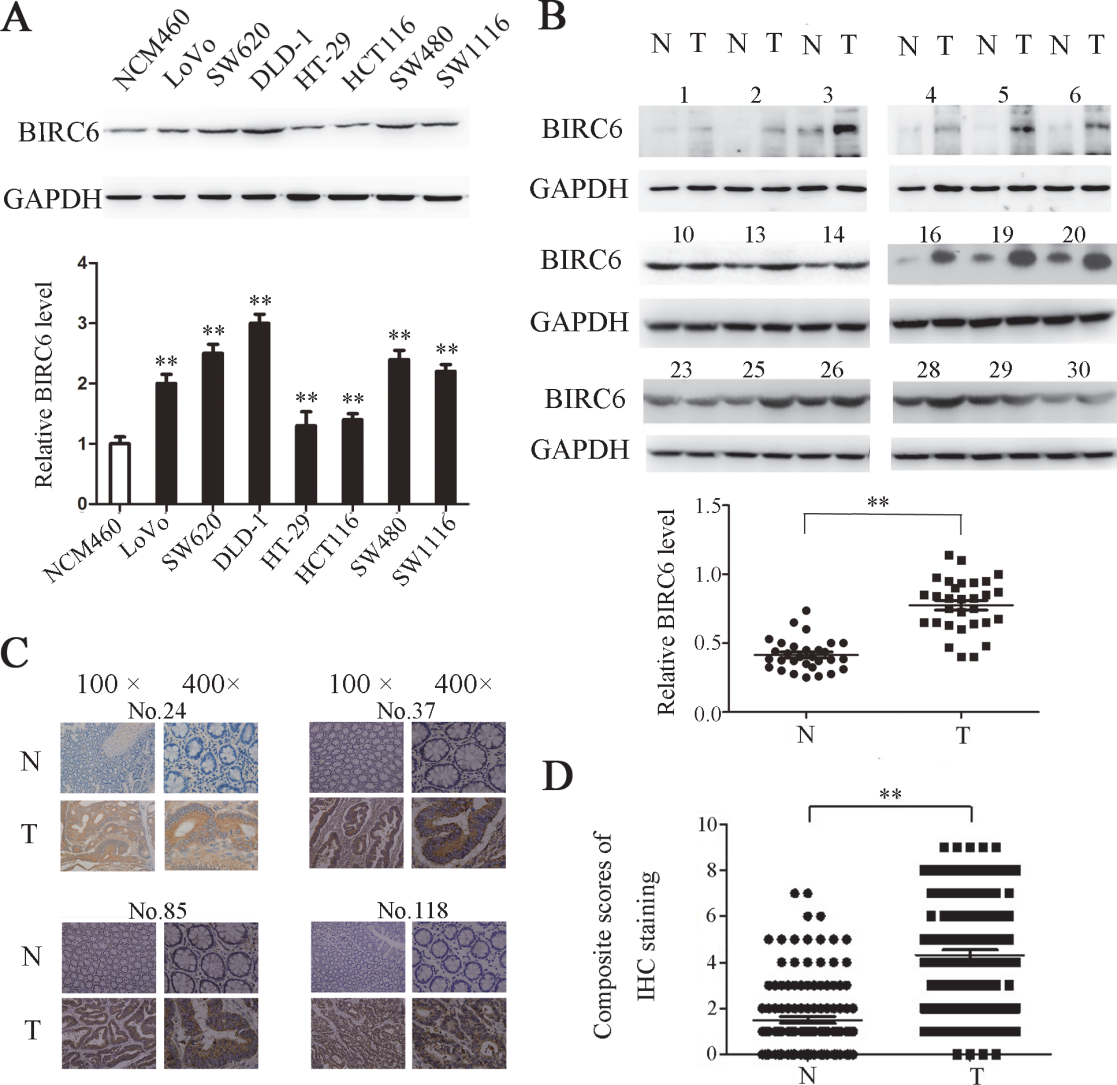
BIRC6 was overexpressed in CRC cell lines and tumor tissues of CRC patients. (A) Expression of BIRC6 in 1 human colon healthy cell line and 7 CRC cell lines. Upper panel: Typical patterns of BIRC6 expression in NCM460 and CRC cell lines. Lower panel: Relative intensity of BIRC6 normalized to GAPDH. Data present Mean ± SEM from three independent experiments. (B) Expression of BIRC6 in 30 paired CRC tissues (T) and adjacent nontumorous tissues (N). Upper panel: Typical patterns of BIRC6 expression in CRC tissues. Lower panel: Relative intensity of BIRC6 normalized to GAPDH. (C) Immunohistochemistry staining of BIRC6 in tumor tissues and self-paired adjacent non-tumorous tissues from four representative CRC patients. (D) Scores of immunohistochemistry staining of BIRC6 in 126 cases. **, *P* < 0.01.

We first detected the BIRC6 expression in 7 CRC cells. Western blotting showed that BIRC6 was overexpressed in LoVo, SW620, DLD-1, HT-29, HCT116, SW480 and SW1116, whereas it was weakly detected in normal colonic epithelial cell line NCM460 (Fig 1A). We next performed Western blotting to examine the BIRC6 expression in 30 paired CRC tissues and adjacent nontumorous tissues. The data implied that BIRC6 was elevated in tumor tissues (Fig 1B). We further assessed the BIRC6 expression in 126 CRC patients by immunohistochemistry. As a result, significant BIRC6 staining was detected in CRC tissues (positive, 73 of 126), whereas the staining in corresponding normal tissues was much weaker (positive, 17 of 126) (Fig 1C and 1D). Notably, the reproducibility of our classification of BIRC6 expression was found to be ‘almost perfect’ (κ-value, 0.816) when the 126 slides of CRC tissues were assessed by two independent observers. The results above indicated that BIRC6 was significantly upregulated in CRC cells lines and clinic CRC tissues.

### Correlation between BIRC6 expression and clinical pathological data

We investigated the correlation of BIRC6 expression with clinicopathologic features in 126 CRC patients. Patient clinical characteristics are listed in S1 Table. There was no significant correlation between BIRC6 expression and age, gender, tumor location, lymph node metastasis (N stage), distant metastasis (M stage), histology type, degree of differentiation, KRAS status and MSI status. However, BIRC6 expression positively correlated with tumor size (*P* = 0.044) and invasion depth (T stage) (*P* = 0.013) (Table 1).

**Table 1:** Correlation between BIRC6 expression and clinicopathologic characteristics.

characteristics		BIRC6 expression in tumor tissues		
		Negative (n = 53)	Positive (n = 73)	*P*
Age (years)	≤50	16	18	0.490
	>50	37	55	
Gender	male	30	41	0.961
	female	23	32	
Tumor location	colon	27	45	0.231
	rectal	26	28	
Tumor size (cm)	≤5cm	49	58	**0.044**
	>5cm	4	15	
Invasion depth	T1-T2	15	8	**0.013**
	T3-T4	38	65	
Lymph node metastasis	N0	30	42	0.917
	N1-N2	23	31	
Distant metastasis	M0	44	58	0.615
	M1	9	15	
TNM stage	I-II	26	36	0.977
	III-IV	27	37	
Tumor histology type	adenocarcinoma	47	61	0.418
	mucinous/signet-ring	6	12	
Tumor degree of differentiation	well/moderate	38	44	0.184
	poor	15	29	
KRAS status	Wild type	39	50	0.54
	Mutation	14	23	
MSI status	MSS	45	59	0.55
	MSI	8	14	

Pearson χ2 tests for all analyses. Bold items have been considered statistically significant. Abbreviations: TNM, tumor-node-metastasis; MSI, microsatellite instability; MSS, microsatellite stable.

### Prognostic value of enhanced BIRC6 expression

**Fig 2:**
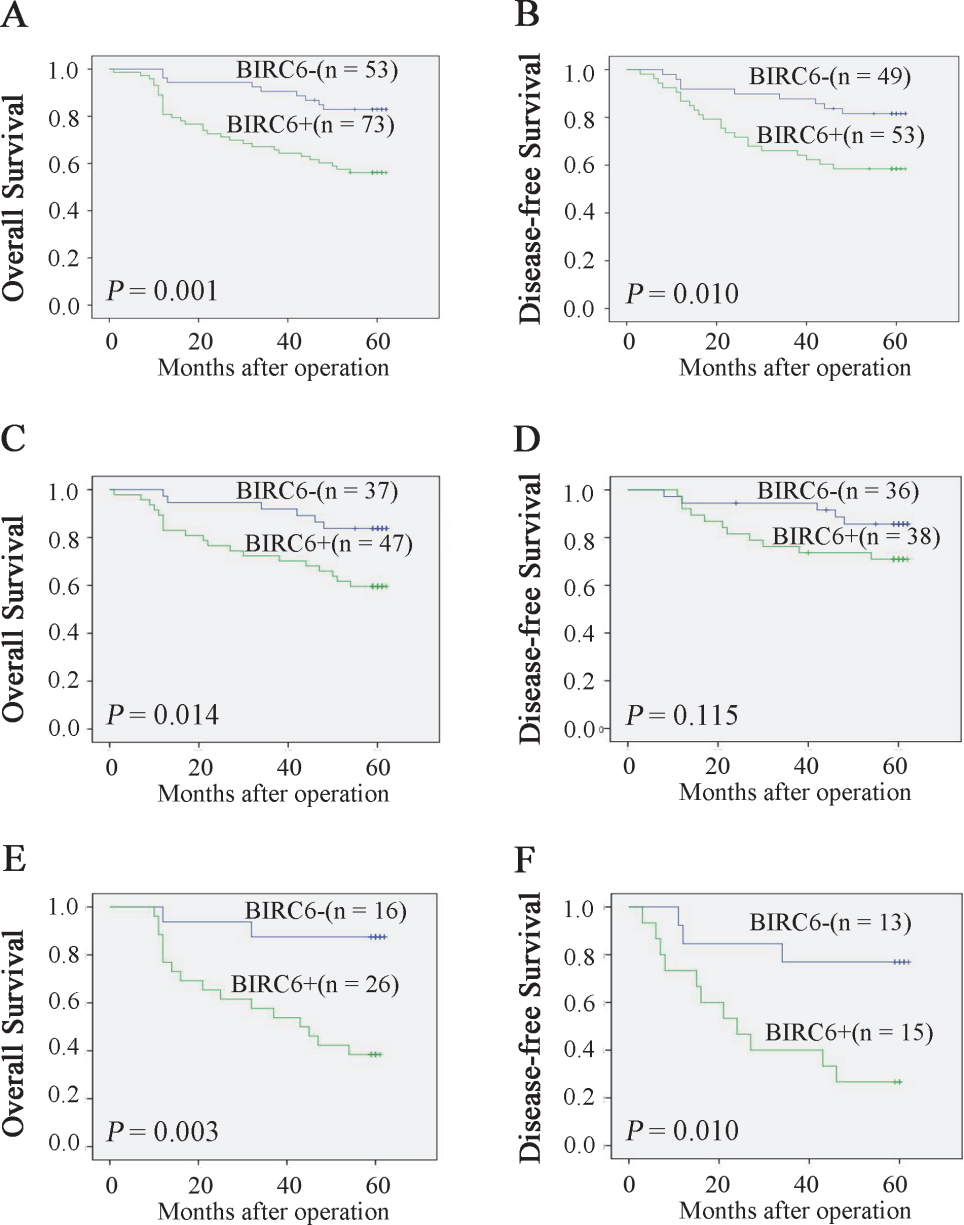
High expression of BIRC6 correlated with poor survival rate. Overall survival (A) and disease-free survival (B) between patients with positive and negative expression of BIRC6 were estimated using the Kaplan-Meier method and compared by the log rank test. Overall survival (C) and disease-free survival (D) were estimated in no chemotherapy group. Overall survival (E) and disease-free survival (F) were estimated in chemotherapy group. Patients with TNM stage IV tumors were excluded when analyzing disease-free survival.

Kaplan-Meier analysis and log-rank test were used to determine the relationship between BIRC6 expression and prognosis. CRC patients with positive BIRC6 expression tended to have shorter overall survival (OS) and disease-free survival (DFS) (*P* = 0.001 and *P* = 0.010, respectively) (Fig 2A and 2B). We next divided patients into two groups: no chemotherapy group and chemotherapy group. As it showed in Fig 2C and 2D, BIRC6 expression was correlated with OS (*P* = 0.038) and DFS (*P* = 0.041) in no chemotherapy group. Similar results were observed in chemotherapy group (*P* = 0.003 and *P* = 0.010) (Fig 2E and 2F). Univariate analysis demonstrated that positive BIRC6 expression was associated with worse OS (*P* = 0.002) and DFS (*P* = 0.013) (Table 2). Other factors correlated with OS were T stage, N stage, M stage and tumor degree of differentiation. Factors affecting DFS included T stage, N stage, KRAS status and MSI status. In addition, multivariate analysis identified enhanced BIRC6 level a risk factor for both OS (*P* = 0.045) and DFS (*P* = 0.026) (Table 3).

**Table 2:** Univariate analysis of factors associated with survival and recurrence.

Variables	OS		DFS	
	Hazard ratio (95% CI)	*P*	Hazard ratio (95% CI)	*P*
Age, y (>50 vs. ≤50)	0.985 (0.502–1.931)	0.964	0.762 (0.359–1.619)	0.479
Gender (female vs. male)	1.405 (0.761–2.594)	0.276	1.531 (0.755–3.107)	0.238
Location (rectal vs. colon)	1.039 (0.561–1.925)	0.903	1.182 (0.584–2.391)	0.642
Tumor size, cm (>5 vs. ≤5)	0.596 (0.212–1.672)	0.325	0.487 (0.148–1.601)	0.236
Invasion depth (T3-T4 vs. T1-T2)	3.174 (0.979–10.285)	0.054	2.084 (0.729–5.959)	0.171
Lymph node metastasis (N1-N2 vs. N0)	3.162 (1.656–6.039)	**0.000**	2.585 (1.265–5.283)	**0.009**
Distant metastasis (M1 vs. M0)	5.977 (3.216–11.106)	**0.000**		
TNM stage (III-IV vs. I-II)	7.573 (3.178–18.048)	**0.000**		
Tumor histology type (adenocarcinoma vs. mucinous/signet-ring)	1.263 (0.560–2.850)	0.574	0.451 (0.108–1.890)	0.276
Tumor degree of differentiation (poor vs. well/moderate)	1.923 (1.041–3.554)	**0.037**	1.122 (0.528–2.382)	0.765
KRAS status (mutation vs. wild type)	1.128 (0.584–2.178)	0.720	2.037 (0.997–4.160)	0.051
MSI status (MSI vs. MSS)	1.774	0.115	2.724	**0.028**
	(0.869–3.620)		(1.114–6.657)	
Expression of BIRC6 (positive vs. negative)	3.170 (1.512–6.647)	**0.002**	2.673 (1.230–5.812)	**0.013**

Bold items have been considered statistically significant. Cox proportional hazards regression model was used in univariate analysis. Patients with TNM stage IV tumors were excluded when analyzing disease-free survival. Abbreviations: 95% CI, 95% confidence interval; OS, overall survival; DFS, disease-free survival; TNM, tumor-node-metastasis; MSI, microsatellite instability; MSS, microsatellite stable.

**Table 3:** Multivariate analysis of factors associated with OS and DFS.

	Hazard ratio (95% CI)	*P*
OS		
Invasion depth (T3-T4 vs. T1-T2)	0.965 (0.270–3.451)	0.956
Lymph node metastasis (N1-N2 vs. N0)	2.406 (1.234–4.692)	**0.010**
Distant metastasis (M1 vs. M0)	4.288 (2.243–8.196)	**0.000**
Tumor degree of differentiation (poor vs. well/moderate)	1.171 (0.621–2.208)	0.625
Expression of BIRC6 (positive vs. negative)	2.214 (1.019–4.810)	**0.045**
DFS		
Invasion depth (T3-T4 vs. T1-T2)	1.028 (0.324–3.260)	0.963
Lymph node metastasis (N1-N2 vs. N0) KRAS status (mutation vs. wild type) MSI status (MSI vs. MSS)	2.198 (1.036–4.666) 1.743 (0.840–3.619) 3.138 (1.259–7.825)	**0.040** 0.136 **0.014**
Expression of BIRC6 (positive vs. negative)	2.564 (1.117–5.886)	**0.026**

Bold items have been considered statistically significant. Multivariate analysis and Cox proportional hazards regression model were used. Variables were adopted for their prognostic significance by univariate analysis (*P* < 0.2). Abbreviations: 95% CI, 95% confidence interval; OS, overall survival; DFS, disease-free survival; MSI, microsatellite instability; MSS, microsatellite stable.

### BIRC6 knockdown inhibited CRC cell proliferation

**Fig 3:**
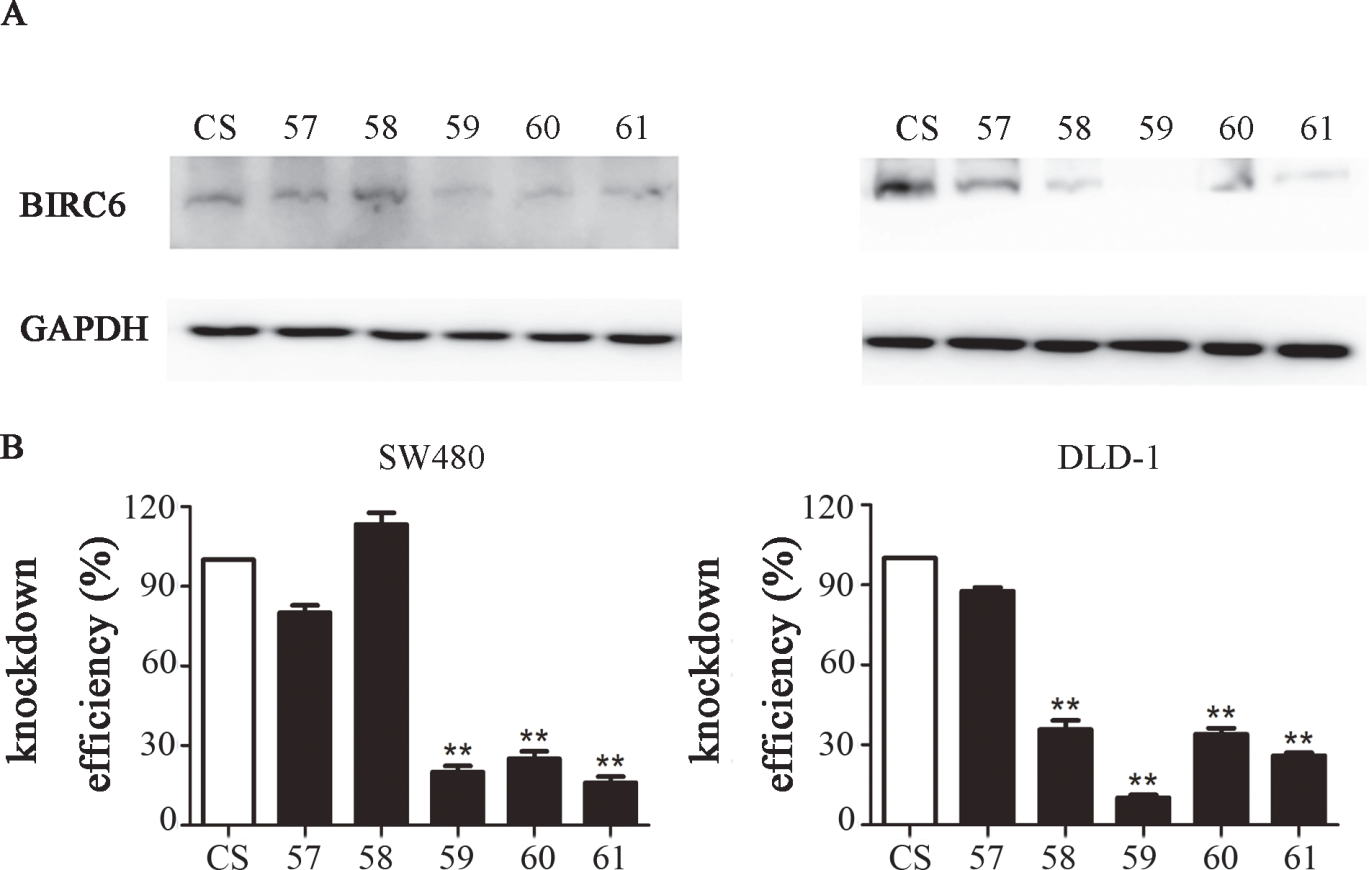
BIRC6 knockdown stable clones were established by lentiviral transduction. (A) BIRC6 knockdown efficiency was testified by Western blotting. (B) Relative expression of BIRC6 normalized to GAPDH was calculated. **, *P* < 0.01 vs. control.

Since the full-length cDNA of BIRC6 extends for 14.5 kb, it is difficult to overexpress BIRC6 in a cell line. Instead, we used lentiviral transduction to establish BIRC6 knockdown stable clones in two CRC cell lines: SW480 and DLD-1. The down-regulated BIRC6 expression was observed significantly in two BIRC6-knockdown cell lines (59 and 61), as shown by Western blotting (Fig 3). These two clones were used in the subsequent analysis.

**Fig 4:**
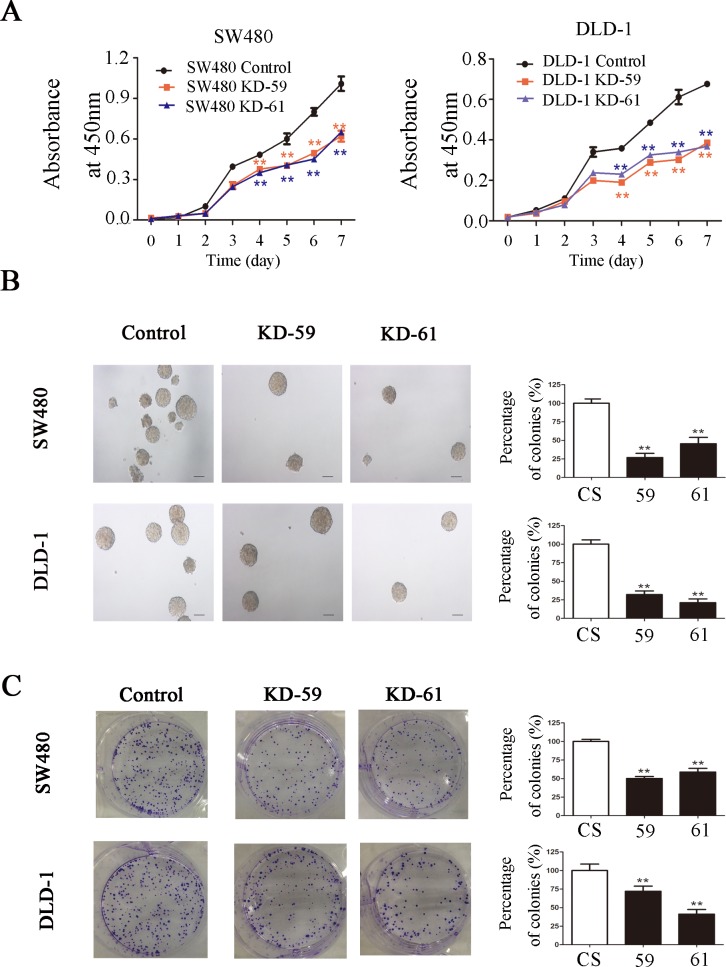
BIRC6 knockdown inhibited CRC cell proliferation. (A) Cell-growth curves of control (CS) and two BIRC6 knockdown clones (59 and 61). (B) Representative images of soft agar colony formation assay and relative levels of colonies in BIRC6 knockdown clones normalized to the control. (C) Representative images of plate colony formation assay and relative levels of colonies in BIRC6 knockdown clones normalized to the control. Data present Mean ± SEM from three independent experiments. **, *P* < 0.01 vs. control.

CCK-8 assay showed that BIRC6 knockdown significantly inhibited the proliferation of SW480 and DLD-1 cells in a time-dependent manner *in vitro* (Fig 4A). Colony formation assay showed that BIRC6 knockdown reduced colony formation in agar in SW480 and DLD-1 cells (Fig 4B). Similar results were observed in colony formation in plates (Fig 4C).

### BIRC6 knockdown induced cell cycle arrest at S phase and downregulated cyclin A2, B1, D1 and E1 levels in CRC cells

**Fig 5:**
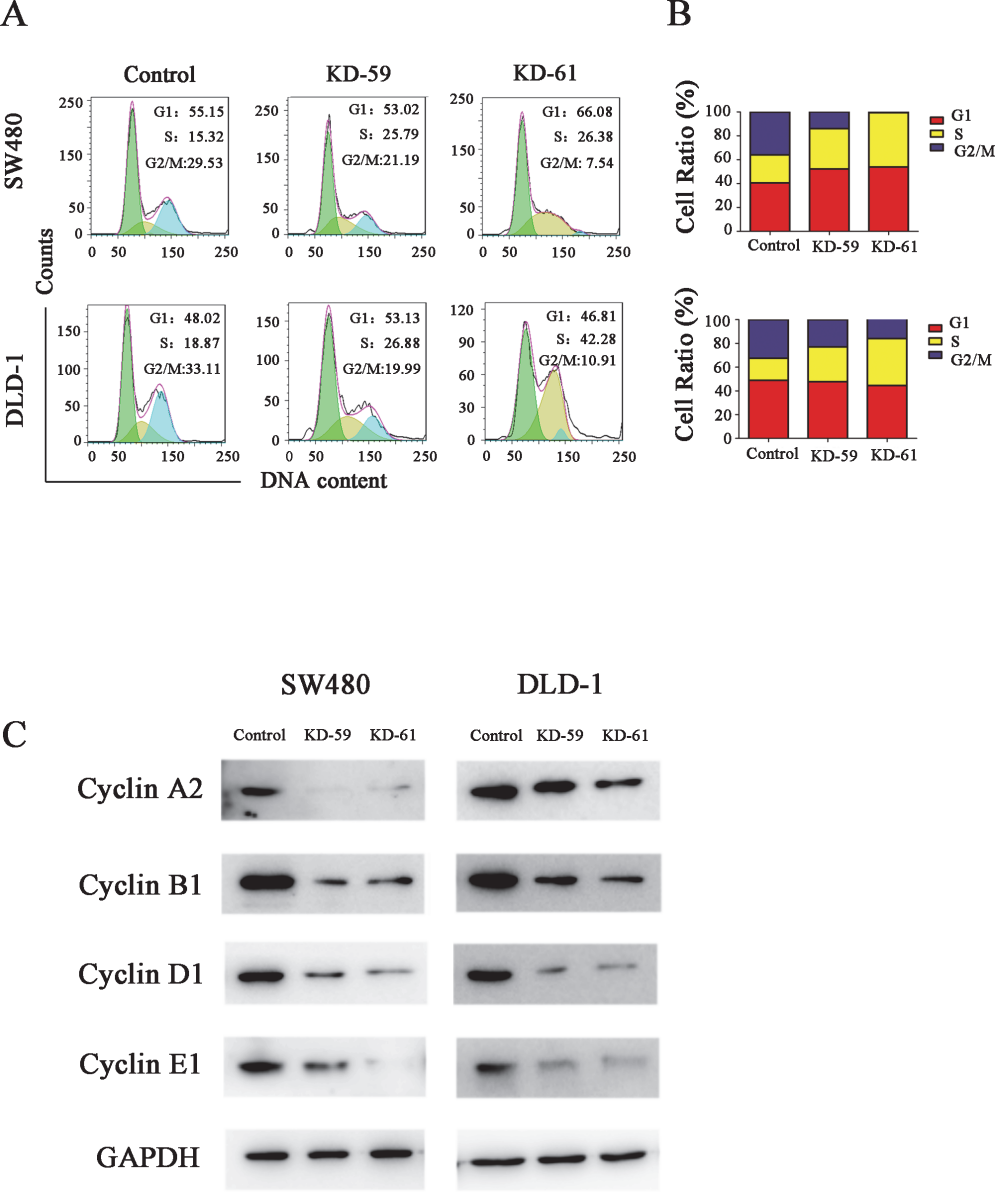
BIRC6 knockdown induced cell cycle arrest at S phase. (A) Representative images of cell cycle assessed by PI staining. (B) Quantification of the cell cycle distribution. Control cells were cells transfected with pLKO.1-puro empty vector; KD-59 and KD-61 were cells transfected with shRNA lentiviral vectors differed in BIRC6-targeting sequences. (C) Expression of cyclin A2, B1, D1 and E1 were performed by Western blotting in the control and two knockdown clones.

Cell cycle analysis showed that the percentage of cells in S phase increased while the percentage of cells in G2/M phase decreased after BIRC6 knockdown in both SW480 cells and DLD-1 cells (Fig 5A and 5B), which indicated that BIRC6 knockdown prevented the CRC cells from entering the mitotic phase. To check whether BIRC6 could regulate the genes involved in cell-cycle control, we detected the effect of BIRC6 on the expression of several cyclins. Western blotting indicated that BIRC6 knockdown decreased the levels of cyclin A2, B1, D1 and E1 in both SW480 cells and DLD-1 cells (Fig 5C).

### BIRC6 knockdown sensitized CRC cells to chemotherapy *in vitro* and *in vivo*

**Fig 6:**
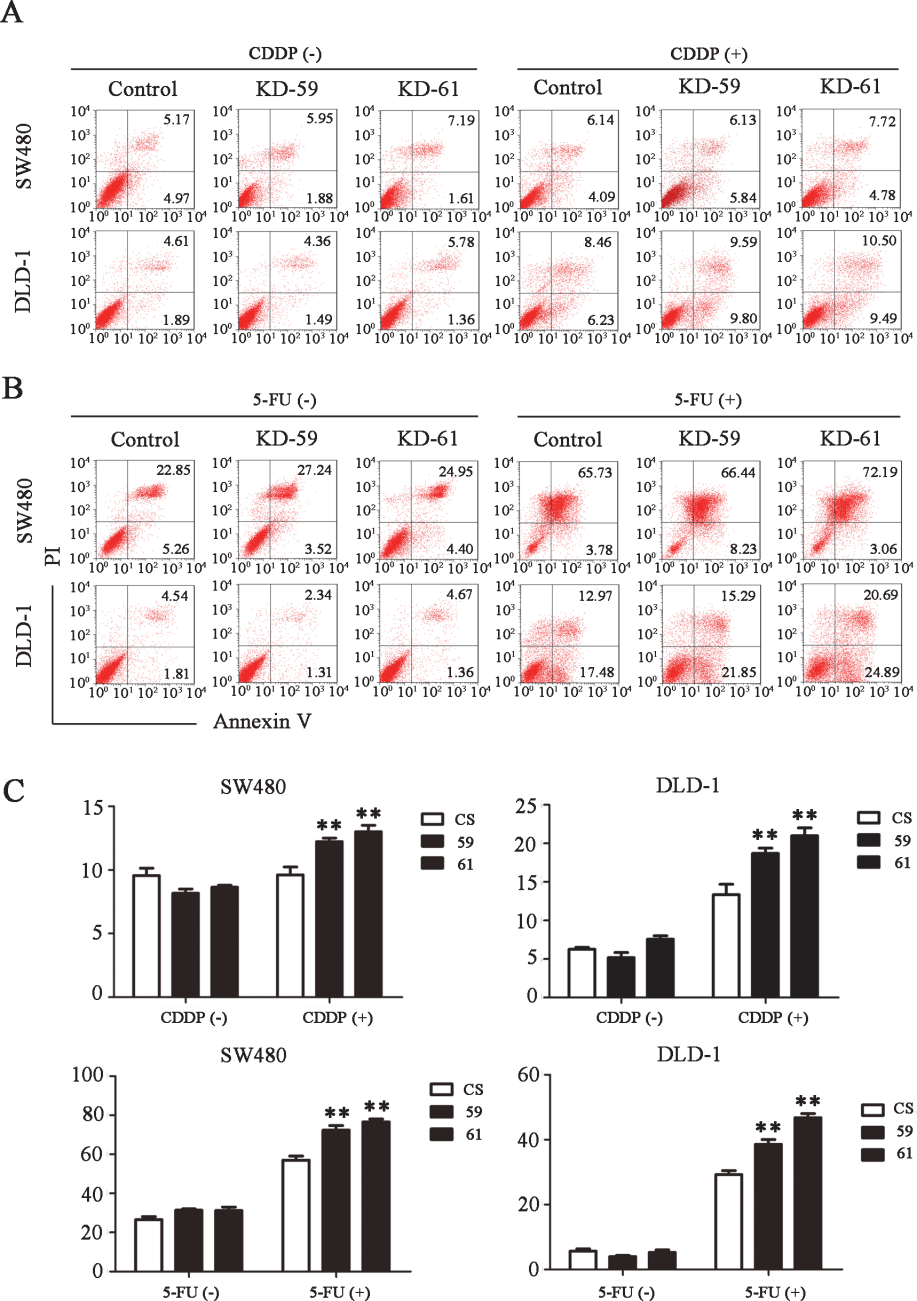
BIRC6 knockdown sensitized CRC cells to cisplatin (CDDP) or 5-fluorouracil (5-FU)-induced apoptosis. (A) Representative images of cell apoptosis assessed by Annexin V/PI staining in the control (CS) and BIRC6 knockdown cells treated with CDDP (40 μM for SW480 or 100 μM for DLD-1) for 24 h or not. (B) Representative images of cell apoptosis for the control and BIRC6 knockdown cells treated with 5-FU (75 μg/mL for SW480 or 50 μg/mL for DLD-1) for 48 h or not. (C) Quantification of the apoptosis rate. Data present Mean ± SEM from three independent experiments. **, *P* < 0.01 vs. control.

**Fig 7:**
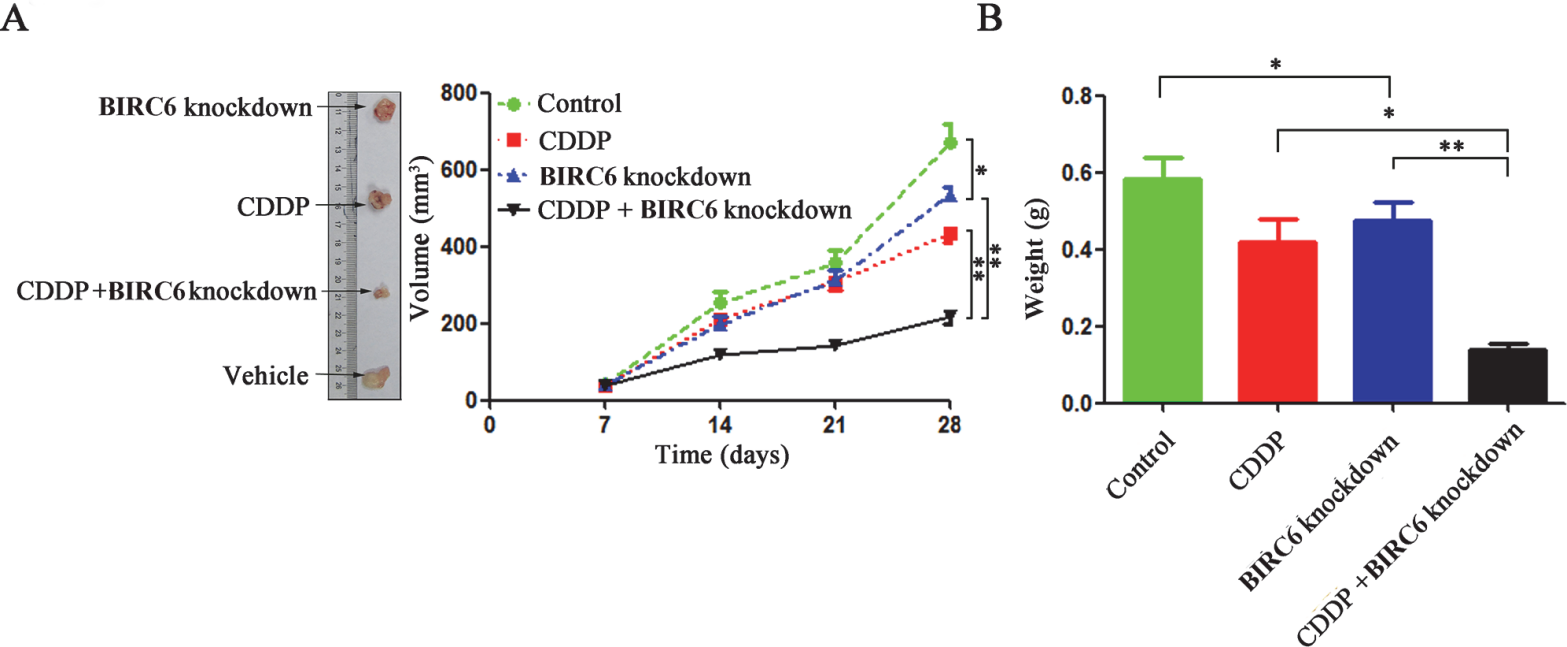
BIRC6 knockdown sensitized CRC cells to chemotherapy in a xenograft nude mouse model. DLD-1 cells stably transfected with control shRNA and BIRC6 knockdown shRNA (59) were injected subcutaneously into nude mice. Tumorbearing mice were treated with CDDP (4 mg/kg; i.p., twice weekly). (A) The tumor size was monitored. (B) Total tumor weight of each group of mice was recorded. Data present Mean ± SEM of 5 mice of each group. *, *P* < 0.05; **, *P* < 0.01.

We investigate the role of BIRC6 in chmosensitivity of ESCC cells. Though BIRC6 knockdown alone did not induce apoptosis in SW480 cells and DLD-1 cells, BIRC6 knockdown sensitized SW480 cells and DLD-1 cells to chemo-induced apoptosis including 5-FU, CDDP, oxaliplatin and CPT-11 (Figs 6A and 6B and S2). The finding that BIRC6 knockdown inhibited cell growth and sensitized CRC cells to chemo-induced apoptosis *in vitro* prompted us to determine whether it exerts a similar effect *in vivo*. Control or BIRC6 stable knockdown clones of DLD-1 were subcutaneously inoculated into nude mice. When all animals had established tumors averaging from 50 to 100 mm^3^, tumorbearing mice were treated with CDDP (4 mg/kg; i.p., twice weekly)[27]. The antitumor efficacy was measured by monitoring the tumor volume and weight after treatment. BIRC6 knockdown slightly inhibited tumor growth. Interestingly, combination of BIRC6 knockdown and CDDP treatment significantly inhibited the tumor growth compared with either BIRC6 knockdown or CDDP treatment alone (Fig 7A and 7B).

## Discussion

Acquired resistance to apoptosis is one of the defining hallmarks of cancer[28]. IAPs, a group of anti-apoptosis proteins that are highly evolutionarily conserved across several species, are key regulators of apoptosis[29]. BIRC6, also named as Apollon, is the largest member of the IAP family with 4830 amino acid residues[12]. It is also the only known IAP member which is membrane associated and localizes to the Golgi compartment and the vesicular system[30]. BIRC6 is highly conserved in different species, suggesting a common biological function. Sequence analysis showed that BIRC6 shared about 92% amino acid identity with Bruce, a member of IAP family from mouse[12]. In addition, there exists the Drosophila homolog of BIRC6 called dBruce[31]. Unlike most of the other mammalian IAPs, BIRC6 have been shown to be essential for viability. Complete inactivation of the Bruce gene led to perinatal lethality and growth retardation in mice embryos after embryonic day 14[32]. Likewise, deletion of the C-terminal half of Bruce caused activation of caspases and apoptosis in the placenta and yolk sac, and resulted in embryonic lethality[17]. Moreover, plenty of evidence indicated that BIRC6 was overexpressed in a variety of cancers including glioma, childhood *de novo* acute myeloid leukemia, breast cancer, neuroblastoma, prostate cancer and non-small-cell lung cancer, and overexpression of BIRC6 was correlated with carcinogenesis, progression and poor prognosis of malignant tumor[12,16,18,33,34,35]. Although the previous study demonstrated that BIRC6 was up-regulated in colorectal cancer using cDNA microarray and qRT-PCR analysis, the relationship between BIRC6 expression and colorectal cancer progression has not been extensively studied.

In the present study, we observed that the expression of BIRC6 could be detected in both colorectal cancer and adjacent tissues but with different percentage. The level of BIRC6 was obviously upregulated in CRC tissues compared with adjacent nontumorous tissues. We also demonstrated that high expression of BIRC6 was associated with adverse clinical and pathologic features in colorectal cancer. Moreover, patients with positive BIRC6 expression tended to survive shorter and had a higher risk of recurrence than those with negative BIRC6 expression. Univariate and multivariate analysis showed that BIRC6 expression was significantly correlated with OS and DFS, indicating that BIRC6 was a predictor of prognosis.

Similarly, BIRC6 was also overexpressed in 7 CRC cell lines. We applied RNAi approach to suppress BIRC6 expression and explored the role of BIRC6 in CRC progression. Our results showed that knockdown of BIRC6 reduced CRC cell proliferation, which is consistent with the previous study in several other tumors. The cell cycle analysis indicated that BIRC6 knockdown resulted in S phase block, thus preventing CRC cells from entering into mitosis. Moreover, We found that BIRC6 knockdown decreased cyclin A2, B1, D1 and E1 levels in CRC cells. We proposed that decreased cyclins levels may contribute to BIRC6 knockdown-induced S phase block. Consistent with our hypothesis, Lee *et al*. [36] found that compound which consist of hinokitiol decreased the levels of cyclin A and cyclin E and led to S phase arrest in HCT116 and SW620 cells. Joe *et al*. [37] reported that resveratrol decreased the levels of cyclins D1, A, and B1 and resulted in S phase arrest in SW480 cells. However, unlike our results, Kikuchi *et al*. [38] found that BIRC6 ubiquitylated cyclin A and BIRC6 deficiency accumulated more cyclin A in 293T cells. Accordingly, the effect of BIRC6 on cyclins appears to depend on cell types and target proteins. Finally, we found that BIRC6 knockdown sensitized CRC cells to chemotherapy both *in vitro* and *in vivo*, which provides a rationale for combining BIRC6 antagonism with chemotherapy to treat CRC.

The present study has certain limitations. For example, the correlation between BIRC6 and prognosis was determined in a limited number of CRC patients (n = 126), and this correlation required confirmation on a larger-scale basis. In the same sense, the fact that we did not observe a significant correlation between BIRC6 expression and MSI status needs further investigation in larger samples before firmer conclusions can be drawn. It's worth noting that we found that BIRC6 regulated cell cycle progression via modulation of cell-cycle-regulation proteins. While increasing evidence have suggested a biological correlation between MSI and alterations in cell-cycle-regulation proteins in cancer[39,40].

In conclusion, the present study provides evidence that BIRC6 functions as a prognostic factor of human CRC. Moreover, our results suggest that BIRC6 knockdown in combination with chemotherapy may have therapeutic potential in the treatment of human CRC.

## Supporting Information

S1 Dataset Data for all Western blotting, immunohistochemistry and survival. (DOCX)

S2 Dataset Data for cell proliferation assay, colony formation assay, cell cycle and apoptosis assay and tumor xenograft models. (DOCX)

S1 Fig Typical patterns of different staining intensity in tumor tissues and peritumoral tissues. Representative images of immunohistochemistry.
(TIF)

S2 Fig BIRC6 knockdown sensitized CRC cells to oxaliplatin or CPT-11-induced apoptosis. (A) Representative images of cell apoptosis assessed by Annexin V/PI staining in the control (CS) and BIRC6 knockdown cells treated with oxaliplatin (10 μM for SW480 and DLD-1) for 48 h or not. (B) Representative images of cell apoptosis for the control and BIRC6 knockdown cells treated with CPT-11 (4 μM for SW480 and DLD-1) for 24 h or not. (C) Quantification of the apoptosis rate. Data present Mean ± SEM from three independent experiments. *, *P* < 0.05; **, *P* < 0.01 vs. control.
(TIF)

S1 Table Characteristics of 126 CRC patients. (DOCX)
