# Supplementary material for: Overexpression of BIRC6 Is a Predictor of Prognosis for Colorectal Cancer
Source: PLoS One. 2015 May 1;10(5):e0125281. doi: 10.1371/journal.pone.0125281 (PMC4416929; doi:10.1371/journal.pone.0125281)
Supplement: S1 Dataset — (DOCX) [file pone.0125281.s001.docx]

Supporting Information Data S1:

Relative BIRC6 expression in several CRC cell lines and human colon healthy cell line.

| NCM460 | 0.8 | 1.2 | 1 |
| --- | --- | --- | --- |
| LoVo | 2.1 | 2.2 | 1.7 |
| SW620 | 2.2 | 2.6 | 2.7 |
| DLD-1 | 3.1 | 3.2 | 2.7 |
| HT-29 | 0.9 | 1.3 | 1.7 |
| HCT116 | 1.5 | 1.5 | 1.2 |
| SW480 | 2.6 | 2.5 | 2.1 |
| SW1116 | 2 | 2.4 | 2.2 |

Relative BIRC6 expression in thirty paired CRC tissues and adjacent nontumorous tissues.

|  | N | T |
| --- | --- | --- |
| 1 | 0.385 | 0.47 |
| 2 | 0.325 | 0.63 |
| 3 | 0.735 | 1.14 |
| 4 | 0.3 | 0.65 |
| 5 | 0.275 | 0.935 |
| 6 | 0.385 | 0.84 |
| 7 | 0.5 | 0.4 |
| 8 | 0.4 | 0.9 |
| 9 | 0.35 | 0.85 |
| 10 | 0.325 | 0.94 |
| 11 | 0.275 | 0.4 |
| 12 | 0.6 | 0.825 |
| 13 | 0.65 | 0.675 |
| 14 | 0.475 | 0.75 |
| 15 | 0.25 | 0.725 |
| 16 | 0.425 | 0.64 |
| 17 | 0.26 | 0.65 |
| 18 | 0.375 | 1.1 |
| 19 | 0.31 | 0.95 |
| 20 | 0.44 | 0.975 |
| 21 | 0.37 | 0.48 |
| 22 | 0.4 | 0.82 |
| 23 | 0.38 | 0.65 |
| 24 | 0.45 | 1 |
| 25 | 0.5 | 0.85 |
| 26 | 0.37 | 0.75 |
| 27 | 0.435 | 0.825 |
| 28 | 0.5 | 0.6 |
| 29 | 0.53 | 0.87 |
| 30 | 0.45 | 0.95 |

immunohistochemistry scores and survival

| Number | Pathology Number | Tumor(T) | | | | | Total | Average | Final Score |  | Non-tumorous(N) | | | | | Total | Average | Final Score | OS（M） | DFS(M) |
| --- | --- | --- | --- | --- | --- | --- | --- | --- | --- | --- | --- | --- | --- | --- | --- | --- | --- | --- | --- | --- |
| 1 | 21020 | 3x3 | 2x3 | 3x3 | 3x3 | 2x3 | 39 | 7.8 | 8 |  | 2x3 | 1x3 | 2x3 | 2x3 | 1x3 | 24 | 4.8 | 5 | 16 |  |
| 2 | 12708 | 3X3 | 1X2 | 1X1 | 3X3 | 3X3 | 30 | 6 | 6 |  | 0 | 0 | 0 | 0 | 0 | 0 | 0 | 0 | 60+ | 60+ |
| 3 | 6752 | 0 | 0 | 0 | 1x3 | 1x3 | 6 | 1.2 | 1 |  | 2x2 | 2x2 | 3x3 | 2x3 | 2x3 | 29 | 5.8 | 6 | 61+ | 61+ |
| 4 | 14501 | 2X3 | 2X3 | 2X3 | 2X3 | 2X3 | 30 | 6 | 6 |  | 1X2 | 1X3 | 1X1 | 1X1 | 1X2 | 9 | 1.8 | 2 | 60+ | 60+ |
| 5 | 15264 | 2X3 | 0 | 0 | 0 | 1X2 | 8 | 1.6 | 2 |  | 0 | 1X3 | 1X3 | 1X1 | 1X2 | 9 | 1.8 | 2 | 44 | 27 |
| 6 | 20995 | 1X2 | 1X3 | 1X3 | 1X3 | 1X2 | 13 | 2.6 | 3 |  | 1X2 | 1X2 | 1X3 | 1X3 | 1X2 | 12 | 2.4 | 2 | 59+ | 59+ |
| 7 | 21288 | 3X3 | 3X3 | 2X3 | 1X3 | 2X3 | 33 | 6.6 | 7 |  | 0 | 1X1 | 1X1 | 0 | 1X1 | 3 | 0.6 | 1 | 59+ | 59+ |
| 8 | 13544 | 2x3 | 1x2 | 1x3 | 2x3 | 1x3 | 20 | 4 | 4 |  | 1x3 | 1x3 | 1x3 | 1x2 | 1x1 | 12 | 2.4 | 2 | 30 | 30 |
| 9 | 12488 | 3x3 | 2x3 | 3x3 | 3x3 | 2x3 | 39 | 7.8 | 8 |  | 1X3 | 1X3 | 1X3 | 1X3 | 2X3 | 18 | 3.6 | 4 | 27 | 14 |
| 10 | 11908 | 1x3 | 1x2 | 1x3 | 1x3 | 0 | 11 | 2.2 | 2 |  | 0 | 1x2 | 2x3 | 2x3 | 1x2 | 16 | 3.2 | 3 | 60+ | 60+ |
| 11 | 21286 | 0 | 0 | 0 | 0 | 0 | 0 | 0 | 0 |  | 0 | 0 | 0 | 0 | 0 | 0 | 0 | 0 | 59+ | 59+ |
| 12 | 21317 | 1X2 | 0 | 1X2 | 1X2 | 1X2 | 8 | 1.6 | 2 |  | 1X2 | 1X2 | 1X2 | 1X1 | 1X2 | 9 | 1.8 | 2 | 59+ | 59+ |
| 13 | 12494 | 0 | 0 | 0 | 1X1 | 2X3 | 7 | 1.4 | 1 |  | 1X3 | 0 | 0 | 1X1 | 0 | 4 | 0.8 | 1 | 60+ | 60+ |
| 14 | 13539 | 1X3 | 2X3 | 1X3 | 2X3 | 1X2 | 20 | 4 | 4 |  | 0 | 0 | 0 | 0 | 0 | 0 | 0 | 0 | 60+ | 60+ |
| 15 | 13243 | 3x3 | 2x3 | 2x3 | 2x3 | 1x3 | 30 | 6 | 6 |  | 2x3 | 1x2 | 1x2 | 1x1 | 1x2 | 13 | 2.6 | 3 | 21 | 6 |
| 16 | 12492 | 2X3 | 2X3 | 2X3 | 2X3 | 2X3 | 30 | 6 | 6 |  | 0 | 0 | 0 | 0 | 0 | 0 | 0 | 0 | 60+ | 60+ |
| 17 | 6753 | 2X2 | 2X2 | 1X2 | 2X2 | 2X2 | 18 | 3.6 | 4 |  | 0 | 0 | 0 | 0 | 0 | 0 | 0 | 0 | 61+ | 61+ |
| 18 | 14255 | 1X2 | 1X3 | 1X1 | 1X1 | 1X2 | 9 | 1.8 | 2 |  | 0 | 0 | 1X1 | 0 | 1X2 | 3 | 0.6 | 1 | 60+ | 60+ |
| 19 | 7025 | 1X3 | 0 | 2X3 | 0 | 0 | 9 | 1.8 | 2 |  | 0 | 0 | 0 | 0 | 1X2 | 2 | 0.4 | 0 | 55+ | 55+ |
| 20 | 22782 | 1x3 | 1x3 | 1x3 | 1x3 | 1x3 | 15 | 3 | 3 |  | 1x2 | 1x2 | 1x2 | 0 | 0 | 6 | 1.2 | 1 | 32 |  |
| 21 | 16897 | 3x3 | 3x3 | 3x3 | 3X3 | 3x3 | 45 | 9 | 9 |  | 1x1 | 1x1 | 0 | 0 | 1x1 | 3 | 0.6 | 1 | 54 |  |
| 22 | 17205 | 1x3 | 2x3 | 1x3 | 1x3 | 1x3 | 18 | 3.6 | 4 |  | 0 | 0 | 0 | 0 | 1x1 | 1 | 0.2 | 0 | 45 | 24 |
| 23 | 17176 | 3X3 | 3X3 | 3X3 | 3X3 | 3X3 | 45 | 9 | 9 |  | 1X1 | 2X3 | 0 | 2X3 | 0 | 13 | 2.6 | 3 | 59+ | 59+ |
| 24 | 16892 | 1x2 | 1x2 | 1x3 | 1x2 | 1x1 | 10 | 2 | 2 |  | 0 | 0 | 1x3 | 0 | 1x2 | 5 | 1 | 1 | 50 |  |
| 25 | 17182 | 2x3 | 1x2 | 1x3 | 1x2 | 1x2 | 15 | 3 | 3 |  | 1x3 | 1x3 | 1x3 | 1x3 | 1x3 | 15 | 3 | 3 | 59+ | 59+ |
| 26 | 17486 | 3x3 | 3x3 | 2x3 | 3x3 | 1x3 | 36 | 7.2 | 7 |  | 0 | 0 | 1x1 | 1x1 | 0 | 2 | 0.4 | 0 | 59+ | 16 |
| 27 | 11905 | 1x1 | 2x3 | 2x3 | 2x3 | 1x2 | 21 | 4.2 | 4 |  | 3x3 | 2x2 | 2x3 | 3x3 | 3x3 | 37 | 7.4 | 7 | 60+ | 60+ |
| 28 | 5860 | 2x3 | 1x3 | 1x3 | 2x3 | 2x3 | 24 | 4.8 | 5 |  | 0 | 0 | 0 | 0 | 0 | 0 | 0 | 0 | 61+ | 61+ |
| 29 | 7023 | 2X3 | 3X3 | 3X3 | 3X3 | 3X3 | 42 | 8.4 | 8 |  | 0 | 0 | 0 | 0 | 0 | 0 | 0 | 0 | 61+ | 61+ |
| 30 | 6771 | 0 | 1X1 | 1X2 | 1X3 | 1X2 | 8 | 1.6 | 2 |  | 0 | 0 | 0 | 0 | 0 | 0 | 0 | 0 | 61+ | 21 |
| 31 | 17186 | 2x3 | 2x3 | 2x3 | 2x3 | 2x3 | 30 | 6 | 6 |  | 1x2 | 1x2 | 1x2 | 1x3 | 1x1 | 10 | 2 | 2 | 59+ | 59+ |
| 32 | 16649 | 1x3 | 1x2 | 0 | 0 | 1x1 | 6 | 1.2 | 1 |  | 1x3 | 1x3 | 2x3 | 2x3 | 2x3 | 24 | 4.8 | 5 | 59+ | 59+ |
| 33 | 17184 | 1x2 | 2x2 | 2x3 | 2x3 | 3x3 | 27 | 5.4 | 5 |  | 2x3 | 2x3 | 1x3 | 2x2 | 3x2 | 25 | 5 | 5 | 60+ | 60+ |
| 34 | 11674 | 2x3 | 1x3 | 1x3 | 1x3 | 1x3 | 18 | 3.6 | 4 |  | 1x2 | 1x1 | 1x3 | 2x3 | 2x2 | 16 | 3.2 | 3 | 60+ | 60+ |
| 35 | 16657 | 3x3 | 3x3 | 3x3 | 3x3 | 3x3 | 45 | 9 | 9 |  | 0 | 0 | 0 | 0 | 0 | 0 | 0 | 0 | 59+ |  |
| 36 | 16426 | 2x2 | 2x3 | 3x3 | 2x3 | 3x3 | 34 | 6.8 | 7 |  | 1x3 | 1x2 | 1x1 | 1x2 | 1x2 | 10 | 2 | 2 | 59+ | 59+ |
| 37 | 5863 | 1x3 | 1x2 | 1x3 | 2x3 | 1x3 | 17 | 3.4 | 3 |  | 0 | 0 | 0 | 0 | 0 | 0 | 0 | 0 | 61+ | 61+ |
| 38 | 12711 | 2x3 | 2x3 | 3x3 | 2x3 | 1x2 | 29 | 5.8 | 6 |  | 2x2 | 1x2 | 1x2 | 1x2 | 0 | 10 | 2 | 2 | 32 |  |
| 39 | 14022 | 1X3 | 1X3 | 2X3 | 3X3 | 2X3 | 27 | 5.4 | 5 |  | 0 | 0 | 0 | 0 | 0 | 0 | 0 | 0 | 60+ | 60+ |
| 40 | 13984 | 2X3 | 2X3 | 2X3 | 3X3 | 3X3 | 36 | 7.2 | 7 |  | 0 | 0 | 0 | 0 | 0 | 0 | 0 | 0 | 11 | 8 |
| 41 | 16978 | 1x3 | 1x3 | 2x3 | 1x3 | 1x1 | 16 | 3.2 | 3 |  | 0 | 0 | 0 | 0 | 0 | 0 | 0 | 0 | 10 |  |
| 42 | 16896 | 1X2 | 1X3 | 1X3 | 1X2 | 1X2 | 12 | 2.4 | 2 |  | 1x2 | 1x2 | 0 | 1x1 | 0 | 5 | 1 | 1 | 42 | 42 |
| 43 | 12011 | 2X3 | 2X2 | 3X3 | 1X2 | 2X3 | 27 | 5.4 | 5 |  | 2X3 | 2X3 | 3X3 | 1X2 | 0 | 23 | 4.6 | 5 | 60+ | 60+ |
| 44 | 7033 | 2X3 | 2X3 | 2X3 | 3X3 | 1X1 | 28 | 5.6 | 6 |  | 0 | 0 | 1X2 | 0 | 1X3 | 5 | 1 | 1 | 61+ | 61+ |
| 45 | 6351 | 1x3 | 1x3 | 1x3 | 1x3 | 1x3 | 15 | 3 | 3 |  | 0 | 1x2 | 0 | 0 | 0 | 2 | 0.4 | 0 | 12 | 11 |
| 46 | 16656 | 0 | 0 | 1X3 | 1X3 | 1X3 | 9 | 1.8 | 2 |  | 2X3 | 2X3 | 2X3 | 2X3 | 1X2 | 26 | 5.2 | 5 | 12 | 12 |
| 47 | 16966 | 2x3 | 2x3 | 3x3 | 3x3 | 2x3 | 36 | 7.2 | 7 |  | 1x1 | 1x2 | 1x2 | 1x1 | `1x1 | 7 | 1.4 | 1 | 59+ | 59+ |
| 48 | 16415 | 3x3 | 3x3 | 3x3 | 3x3 | 2x3 | 42 | 8.4 | 8 |  | 2x3 | 2x3 | 1x3 | 1x1 | 1x1 | 17 | 3.4 | 3 | 1 |  |
| 49 | 6107 | 1x2 | 0 | 0 | 1x2 | 1x2 | 6 | 1.2 | 1 |  | 0 | 0 | 0 | 1x1 | 0 | 1 | 0.2 | 0 | 61+ | 61+ |
| 50 | 15035 | 2X3 | 2X3 | 3X3 | 3X3 | 3X3 | 39 | 7.8 | 8 |  | 0 | 0 | 0 | 0 | 1X1 | 1 | 0.2 | 0 | 22 | 22 |
| 51 | 14326 | 1X2 | 1X3 | 1X3 | 1X2 | 1X3 | 13 | 2.6 | 3 |  | 1X1 | 1X1 | 0 | 1X1 | 0 | 3 | 0.6 | 1 | 12 |  |
| 52 | 14761 | 0 | 0 | 0 | 1X1 | 0 | 1 | 0.2 | 0 |  | 1X3 | 1X3 | 1X3 | 1X3 | 1X2 | 14 | 2.8 | 3 | 60+ | 60+ |
| 53 | 12740 | 0 | 1X2 | 1X2 | 1X2 | 0 | 6 | 1.2 | 1 |  | 1X2 | 0 | 1X2 | 0 | 0 | 4 | 0.8 | 1 | 60+ | 60+ |
| 54 | 13238 | 3x3 | 1x1 | 2x3 | 2x3 | 2x3 | 28 | 5.6 | 6 |  | 1x1 | 0 | 1x1 | 1x1 | 0 | 3 | 0.6 | 1 | 14 |  |
| 55 | 12240 | 1X2 | 1X2 | 0 | 1X2 | 0 | 6 | 1.2 | 1 |  | 1X2 | 1X2 | 0 | 0 | 0 | 4 | 0.8 | 1 | 60+ | 44 |
| 56 | 14755 | 2X3 | 1X3 | 1X3 | 2X3 | 2X3 | 24 | 4.8 | 5 |  | 0 | 0 | 0 | 0 | 1X1 | 1 | 0.2 | 0 | 59+ | 59+ |
| 57 | 9351 | 3X3 | 1X2 | 1X3 | 1X3 | 2X3 | 23 | 4.6 | 5 |  | 0 | 1X2 | 0 | 0 | 0 | 2 | 0.4 | 0 | 61+ | 61+ |
| 58 | 14257 | 1X1 | 0 | 2X2 | 2X1 | 0 | 7 | 1.4 | 1 |  | 0 | 0 | 0 | 1X1 | 0 | 1 | 0.2 | 0 | 9 |  |
| 59 | 6123 | 0 | 0 | 1x2 | 0 | 1x2 | 4 | 0.8 | 1 |  | 0 | 0 | 0 | 0 | 0 | 0 | 0 | 0 | 61+ | 61+ |
| 60 | 13545 | 1X2 | 1X3 | 1X2 | 1X2 | 1X1 | 10 | 2 | 2 |  | 0 | 0 | 0 | 0 | 0 | 0 | 0 | 0 | 46 | 46 |
| 61 | 13761 | 1X2 | 1X1 | 1X1 | 0 | 1X2 | 6 | 1.2 | 1 |  | 1X1 | 1X1 | 0 | 0 | 1X1 | 3 | 0.6 | 1 | 60+ | 60+ |
| 62 | 6844 | 2X3 | 1X2 | 1X2 | 3X3 | 1X1 | 20 | 4 | 4 |  | 0 | 0 | 1X1 | 0 | 0 | 1 | 0.2 | 0 | 11 |  |
| 63 | 6108 | 2x2 | 3x3 | 2x3 | 3x3 | 2x3 | 34 | 6.8 | 7 |  | 1x2 | 1x2 | 1x1 | 0 | 1x1 | 6 | 1.2 | 1 | 61+ | 61+ |
| 64 | 13515 | 0 | 1X2 | 1X2 | 1X3 | 1X3 | 10 | 2 | 2 |  | 1X1 | 0 | 0 | 1X1 | 2X3 | 8 | 1.6 | 2 | 12 | 12 |
| 65 | 18053 | 3x3 | 3x3 | 3x3 | 3x3 | 2x3 | 42 | 8.4 | 8 |  | 1x2 | 1x2 | 0 | 0 | 0 | 4 | 0.8 | 1 | 59+ | 59+ |
| 66 | 18032 | 2x3 | 3x3 | 2x3 | 2x3 | 2x3 | 33 | 6.6 | 7 |  | 0 | 0 | 0 | 1x1 | 0 | 1 | 0.2 | 0 | 12 | 7 |
| 67 | 3482 | 1x1 | 1x2 | 0 | 0 | 0 | 3 | 0.6 | 1 |  | 1x2 | 1x2 | 1x2 | 1x1 | 0 | 7 | 1.4 | 1 | 25 |  |
| 68 | 3658 | 1x3 | 1x2 | 2x3 | 2x3 | 2x3 | 23 | 4.6 | 5 |  | 0 | 1x1 | 1x1 | 0 | 1x2 | 4 | 0.8 | 1 | 12 | 12 |
| 69 | 18475 | 1x3 | 1x3 | 2x3 | 2x3 | 1x3 | 21 | 4.2 | 4 |  | 1x2 | 1x2 | 1x2 | 0 | 1x1 | 7 | 1.4 | 1 | 59+ | 59+ |
| 70 | 18322 | 1x1 | 2x2 | 2x3 | 2x3 | 1x2 | 19 | 3.8 | 4 |  | 2x2 | 2x2 | 1x2 | 2x2 | 1x2 | 16 | 3.2 | 3 | 59+ | 59+ |
| 71 | 18695 | 2x3 | 2x3 | 3x3 | 3x3 | 3x3 | 39 | 7.8 | 8 |  | 2x3 | 1x3 | 2x3 | 2x3 | 2x3 | 27 | 5.4 | 5 | 11 | 11 |
| 72 | 15545 | 2X3 | 2X3 | 2X3 | 2X3 | 1X3 | 27 | 5.4 | 5 |  | 1X1 | 2X3 | 1X2 | 2X2 | 3X3 | 22 | 4.4 | 4 | 17 | 17 |
| 73 | 3266 | 1x2 | 1x1 | 1x2 | 1x3 | 1x2 | 10 | 2 | 2 |  | 0 | 0 | 0 | 0 | 1x3 | 3 | 0.6 | 1 | 48 | 48 |
| 74 | 11211 | 0 | 1x2 | 1x1 | 1x2 | 1x2 | 7 | 1.4 | 1 |  | 2x3 | 2x3 | 2x3 | 2x3 | 2x3 | 30 | 6 | 6 | 60+ | 60+ |
| 75 | 3807 | 1X2 | 1X1 | 1X1 | 0 | 0 | 4 | 0.8 | 1 |  | 1X3 | 1X3 | 1X3 | 1X2 | 1X2 | 13 | 2.6 | 3 | 47 | 27 |
| 76 | 11663 | 3X3 | 3X3 | 2X3 | 2X3 | 1X3 | 33 | 6.6 | 7 |  | 1X2 | 0 | 0 | 0 | 1X1 | 3 | 0.6 | 1 | 60+ | 60+ |
| 77 | 11668 | 2x3 | 2x3 | 2x3 | 3x3 | 1x2 | 29 | 5.8 | 6 |  | 1x2 | 1x2 | 1x2 | 1x2 | 1x3 | 11 | 2.2 | 2 | 21 | 21 |
| 78 | 10314 | 1X3 | 1X2 | 1X3 | 1X3 | 1X3 | 14 | 2.8 | 3 |  | 1X3 | 1X3 | 1X3 | 1X2 | 1X3 | 14 | 2.8 | 3 | 61+ | 61+ |
| 79 | 11472 | 1x3 | 2x3 | 2x3 | 3x3 | 2x3 | 30 | 6 | 6 |  | 1x3 | 1x2 | 1x1 | 1x2 | 1x2 | 10 | 2 | 2 | 51 |  |
| 80 | 10581 | 1X2 | 0 | 1X2 | 0 | 1X2 | 6 | 1.2 | 1 |  | 0 | 0 | 1X2 | 1X2 | 0 | 4 | 0.8 | 1 | 61+ | 61+ |
| 81 | 10614 | 2x3 | 2x3 | 2x3 | 2x3 | 3x3 | 33 | 6.6 | 7 |  | 1x1 | 1x1 | 1x1 | 0 | 1x1 | 4 | 0.8 | 1 | 12 |  |
| 82 | 10613 | 2X3 | 2X3 | 1X3 | 1X3 | 2X3 | 24 | 4.8 | 5 |  | 0 | 0 | 0 | 0 | 0 | 0 | 0 | 0 | 61+ | 61+ |
| 83 | 2906 | 1x2 | 1x1 | 1x1 | 1x2 | 1x1 | 7 | 1.4 | 1 |  | 2x3 | 1x3 | 1x3 | 1x1 | 0 | 13 | 2.6 | 3 | 62+ | 62+ |
| 84 | 3273 | 0 | 0 | 0 | 1x1 | 0 | 1 | 0.2 | 0 |  | 0 | 0 | 1x1 | 1x1 | 1x3 | 5 | 1 | 1 | 62+ |  |
| 85 | 2257 | 3x3 | 3x3 | 3x3 | 2x3 | 1x1 | 34 | 6.8 | 7 |  | 0 | 0 | 0 | 0 | 0 | 0 | 0 | 0 | 62+ | 62+ |
| 86 | 16128 | 2x3 | 2x3 | 3x3 | 2x3 | 3x3 | 36 | 7.2 | 7 |  | 0 | 0 | 0 | 1x1 | 1x2 | 3 | 0.6 | 1 | 60+ | 60+ |
| 87 | 2617 | 1x3 | 1x3 | 1x1 | 1x2 | 1x2 | 11 | 2.2 | 2 |  | 0 | 0 | 0 | 0 | 0 | 0 | 0 | 0 | 62+ | 62+ |
| 88 | 14520 | 3x3 | 2x3 | 2x3 | 2x3 | 2x3 | 33 | 6.6 | 7 |  | 0 | 0 | 0 | 0 | 0 | 0 | 0 | 0 | 60+ | 60+ |
| 89 | 2448 | 1x2 | 1x3 | 1x3 | 2x3 | 3x3 | 23 | 4.6 | 5 |  | 0 | 0 | 0 | 0 | 0 | 0 | 0 | 0 | 12 |  |
| 90 | 2638 | 1x1 | 1x1 | 0 | 0 | 1x2 | 4 | 0.8 | 1 |  | 1x2 | 0 | 0 | 1x2 | 1x3 | 7 | 1.4 | 1 | 13 | 8 |
| 91 | 15566 | 3x3 | 2x3 | 1x3 | 2x3 | 2x3 | 30 | 6 | 6 |  | 1x1 | 0 | 1x1 | 0 | 0 | 2 | 0.4 | 0 | 60+ | 46 |
| 92 | 21281 | 2x2 | 2x3 | 2x3 | 1x3 | 2x2 | 23 | 4.6 | 5 |  | 0 | 1x1 | 0 | 1x1 | 0 | 2 | 0.4 | 0 | 59+ | 59+ |
| 93 | 15893 | 1x1 | 0 | 1x1 | 2x3 | 1x3 | 11 | 2.2 | 2 |  | 0 | 1x1 | 0 | 1x1 | 1x1 | 3 | 0.6 | 1 | 60+ |  |
| 94 | 15890 | 0 | 0 | 0 | 0 | 0 | 0 | 0 | 0 |  | 1x2 | 0 | 0 | 0 | 0 | 2 | 0.4 | 0 | 60+ | 60+ |
| 95 | 17209 | 1x2 | 1x1 | 1x3 | 1x3 | 1x2 | 11 | 2.2 | 2 |  | 1x1 | 1x2 | 0 | 1x1 | 1x1 | 5 | 1 | 1 | 59+ | 59+ |
| 96 | 10940 | 3x3 | 2x3 | 2x3 | 2x3 | 1x3 | 30 | 6 | 6 |  | 0 | 0 | 0 | 0 | 0 | 0 | 0 | 0 | 60+ | 60+ |
| 97 | 10916 | 2x3 | 1x3 | 1x3 | 1x2` | 1x2 | 16 | 3.2 | 3 |  | 0 | 0 | 0 | 0 | 1x1 | 1 | 0.2 | 0 | 34 |  |
| 98 | 15269 | 2x3 | 3x3 | 1x3 | 0 | 0 | 18 | 3.6 | 4 |  | 1x2 | 1x2 | 1x2 | 0 | 1x1 | 7 | 1.4 | 1 | 60+ | 60+ |
| 99 | 16394 | 3x3 | 3x3 | 2x3 | 2x3 | 3x3 | 39 | 7.8 | 8 |  | 0 | 0 | 0 | 0 | 0 | 0 | 0 | 0 | 43 | 43 |
| 100 | 14733 | 2x3 | 1x3 | 3x3 | 1x2 | 1x3 | 23 | 4.6 | 5 |  | 0 | 0 | 0 | 0 | 1x1 | 1 | 0.2 | 0 | 60+ | 40 |
| 101 | 18700 | 2x3 | 1x3 | 2x3 | 1x3 | 1x3 | 21 | 4.2 | 4 |  | 1x2 | 1x1 | 1x1 | 1x1 | 0 | 5 | 1 | 1 | 10 |  |
| 102 | 19273 | 3x3 | 3x3 | 3x3 | 2x3 | 2x3 | 39 | 7.8 | 8 |  | 3x2 | 3x2 | 3x2 | 1x2 | 1x1 | 21 | 4.2 | 4 | 7 |  |
| 103 | 15288 | 2x3 | 2x3 | 1x3 | 1x2 | 3x3 | 26 | 5.2 | 5 |  | 2x2 | 1x3 | 2x3 | 2x2 | 2x3 | 23 | 4.6 | 5 | 60+ | 60+ |
| 104 | 11192 | 1x3 | 1x3 | 1x2 | 1x3 | 1x2 | 13 | 2.6 | 3 |  | 2x2 | 1x1 | 1x2 | 1x2 | 1x2 | 11 | 2.2 | 2 | 60+ | 60+ |
| 105 | 14523 | 3x3 | 1x3 | 1x2 | 1x2 | 3x3 | 25 | 5 | 5 |  | 1x3 | 1x2 | 1x1 | 0 | 0 | 6 | 1.2 | 1 | 60+ | 3 |
| 106 | 3642 | 3x3 | 1x2 | 1x2 | 0 | 0 | 13 | 2.6 | 3 |  | 1X1 | 1x2 | 1x1 | 2x2 | 1x2 | 10 | 2 | 2 | 62+ | 62+ |
| 107 | 17464 | 2X2 | 1X3 | 2X3 | 2X3 | 1X2 | 21 | 4.2 | 4 |  | 0 | 1X1 | 2X3 | 0 | 0 | 7 | 1.4 | 1 | 59+ | 59+ |
| 108 | 18028 | 1x1 | 1x3 | 1x2 | 1x3 | 1x1 | 10 | 2 | 2 |  | 1x2 | 1x2 | 0 | 0 | 1x2 | 6 | 1.2 | 1 | 59+ | 59+ |
| 109 | 3632 | 0 | 1X2 | 1X3 | 1X3 | 1X2 | 10 | 2 | 2 |  | 0 | 3X3 | 0 | 1X2 | 1X2 | 13 | 2.6 | 3 | 62+ | 62+ |
| 110 | 18295 | 3X3 | 3X3 | 3X3 | 3X3 | 3X3 | 45 | 9 | 9 |  | 2X3 | 2X3 | 0 | 2X3 | 2X2 | 22 | 4.4 | 4 | 59+ | 59+ |
| 111 | 19275 | 3x3 | 2x3 | 3x3 | 2x3 | 3x3 | 39 | 7.8 | 8 |  | 2x3 | 1x3 | 1x3 | 2x3 | 1x3 | 21 | 4.2 | 4 | 59+ |  |
| 112 | 14023 | 1X3 | 1X2 | 0 | 0 | 0 | 5 | 1 | 1 |  | 0 | 0 | 0 | 0 | 0 | 0 | 0 | 0 | 60+ | 24 |
| 113 | 11204 | 1x3 | 1x3 | 1x3 | 2x3 | 2x3 | 21 | 4.2 | 4 |  | 1x1 | 1x1 | 1x2 | 1x1 | 0 | 5 | 1 | 1 | 60+ |  |
| 114 | 11647 | 3x3 | 3x3 | 3x3 | 3X3 | 3x3 | 45 | 9 | 9 |  | 0 | 0 | 0 | 1x2 | 1x2 | 4 | 0.8 | 1 | 60+ | 60+ |
| 115 | 16393 | 3x3 | 3x3 | 3x3 | 3x3 | 2x3 | 42 | 8.4 | 8 |  | 1x3 | 1x3 | 1x3 | 1x3 | 1x3 | 15 | 3 | 3 | 59+ | 59+ |
| 116 | 10942 | 1x1 | 0 | 1x1 | 1x2 | 0 | 4 | 0.8 | 1 |  | 1x2 | 1x1 | 0 | 1x1 | 0 | 4 | 0.8 | 1 | 60+ | 12 |
| 117 | 10922 | 1x2 | 1x3 | 1x3 | 1x3 | 0 | 11 | 2.2 | 2 |  | 0 | 0 | 0 | 0 | 0 | 0 | 0 | 0 | 60+ | 34 |
| 118 | 18976 | 3x3 | 1x2 | 1x2 | 1x2 | 1x3 | 18 | 3.6 | 4 |  | 0 | 0 | 0 | 0 | 0 | 0 | 0 | 0 | 47 |  |
| 119 | 19268 | 2x3 | 2x3 | 3x3 | 3x3 | 3x3 | 39 | 7.8 | 8 |  | 0 | 0 | 0 | 0 | 0 | 0 | 0 | 0 | 59+ | 59+ |
| 120 | 18982 | 1x3 | 1x3 | 1x2 | 0 | 0 | 8 | 1.6 | 2 |  | 3x3 | 3x3 | 3x3 | 1x2 | 2x2 | 33 | 6.6 | 7 | 37 | 15 |
| 121 | 16160 | 3x3 | 3x3 | 2x3 | 3x3 | 3x3 | 42 | 8.4 | 8 |  | 0 | 0 | 0 | 1x1 | 0 | 1 | 0.2 | 0 | 38 | 38 |
| 122 | 14737 | 1x3 | 1x2 | 1x3 | 2x3 | 1x3 | 17 | 3.4 | 3 |  | 2x3 | 1x3 | 0 | 1x2 | 1x1 | 12 | 2.4 | 2 | 60+ | 60+ |
| 123 | 18975 | 3x3 | 2x3 | 3x3 | 3x3 | 3x3 | 42 | 8.4 | 8 |  | 2x3 | 2x3 | 1x3 | 1x2 | 1x1 | 18 | 3.6 | 4 | 59+ | 59+ |
| 124 | 14522 | 1x3 | 2x3 | 2x3 | 1x3 | 1x3 | 21 | 4.2 | 4 |  | 0 | 0 | 0 | 0 | 0 | 0 | 0 | 0 | 60+ | 60+ |
| 125 | 10345 | 1X3 | 1X2 | 1X2 | 1X2 | 1X3 | 12 | 2.4 | 2 |  | 0 | 0 | 0 | 1X2 | 0 | 2 | 0.4 | 0 | 61+ | 61+ |
| 126 | 11644 | 2X3 | 3X3 | 3X3 | 2X3 | 3X3 | 39 | 7.8 | 8 |  | 1X2 | 1X2 | 1X3 | 1X3 | 1X2 | 12 | 2.4 | 2 | 54 | 54 |
|  |  |  |  |  |  |  |  | 541 |  |  |  |  |  |  |  |  | 191.8 |  |  |  |
|  |  |  |  |  |  |  |  | 4.29 | 4.32±2.579 | |  |  |  |  |  |  | 1.52 | 1.51±1.715 | |  |

Data for all Western blotting, immunohistochemistry and survival.
