# Supplementary material for: Overexpression of BIRC6 Is a Predictor of Prognosis for Colorectal Cancer
Source: PLoS One. 2015 May 1;10(5):e0125281. doi: 10.1371/journal.pone.0125281 (PMC4416929; doi:10.1371/journal.pone.0125281)
Supplement: S2 Dataset — (DOCX) [file pone.0125281.s002.docx]

Supporting Information Data S2:

Cell proliferation assay

| day | SW480 CS | | | SW480 KD-59 | | | SW480 KD-61 | | |
| --- | --- | --- | --- | --- | --- | --- | --- | --- | --- |
| 0 | 0.008 | 0.0093 | 0.0106 | 0.01 | 0.0157 | 0.0214 | 0.007 | 0.008 | 0.009 |
| 1 | 0.015 | 0.0177 | 0.0204 | 0.0226 | 0.0263 | 0.03 | 0.02 | 0.0317 | 0.0434 |
| 2 | 0.09 | 0.1005 | 0.111 | 0.045 | 0.0482 | 0.0514 | 0.045 | 0.0488 | 0.0526 |
| 3 | 0.37 | 0.3942 | 0.4186 | 0.209 | 0.2545 | 0.3 | 0.22 | 0.2452 | 0.2704 |
| 4 | 0.45 | 0.4825 | 0.515 | 0.35 | 0.3768 | 0.4036 | 0.32 | 0.35 | 0.38 |
| 5 | 0.53 | 0.55 | 0.58 | 0.38 | 0.4028 | 0.4256 | 0.38 | 0.4063 | 0.4326 |
| 6 | 0.75 | 0.8 | 0.85 | 0.47 | 0.496 | 0.522 | 0.42 | 0.45 | 0.48 |
| 7 | 0.916 | 1.008 | 1.1 | 0.55 | 0.6202 | 0.6904 | 0.64 | 0.6502 | 0.6604 |
|  |  |  |  |  |  |  |  |  |  |
|  |  |  |  |  |  |  |  |  |  |
| day | DLD-1 CS | | | DLD-1 KD-59 | | | DLD-1 KD-61 | | |
| 0 | 0.018 | 0.02 | 0.017 | 0.016 | 0.02 | 0.024 | 0.015 | 0.018 | 0.021 |
| 1 | 0.053 | 0.0525 | 0.052 | 0.037 | 0.0373 | 0.0376 | 0.0435 | 0.043 | 0.0425 |
| 2 | 0.1002 | 0.1102 | 0.1202 | 0.09 | 0.0962 | 0.1024 | 0.08 | 0.0754 | 0.0808 |
| 3 | 0.3 | 0.3403 | 0.3806 | 0.22 | 0.1996 | 0.1792 | 0.25 | 0.2379 | 0.2258 |
| 4 | 0.34 | 0.3582 | 0.3764 | 0.18 | 0.1903 | 0.2006 | 0.22 | 0.2302 | 0.2404 |
| 5 | 0.47 | 0.4853 | 0.5006 | 0.28 | 0.2888 | 0.2976 | 0.3 | 0.3254 | 0.3508 |
| 6 | 0.5 | 0.612 | 0.724 | 0.3 | 0.3028 | 0.3056 | 0.32 | 0.34 | 0.36 |
| 7 | 0.6 | 0.679 | 0.758 | 0.37 | 0.3859 | 0.4018 | 0.36 | 0.3684 | 0.3768 |

Colony formation assay

| SW480 soft agar colony | CS | 90 | 110 | 100 |
| --- | --- | --- | --- | --- |
|  | KD-59 | 25 | 38 | 18 |
|  | KD-61 | 38 | 63 | 36 |
|  |  |  |  |  |
|  |  |  |  |  |
| DLD-1 soft agar colony | CS | 90 | 110 | 100 |
|  | KD-59 | 22 | 38 | 36 |
|  | KD-61 | 11 | 25 | 27 |
|  |  |  |  |  |
|  |  |  |  |  |
| SW480 plate colony | CS | 95 | 100 | 105 |
|  | KD-59 | 45 | 50 | 55 |
|  | KD-61 | 50 | 59 | 68 |
|  |  |  |  |  |
|  |  |  |  |  |
| DLD-1 plate colony | CS | 84 | 100 | 116 |
|  | KD-59 | 60 | 72 | 84 |
|  | KD-61 | 30 | 42 | 52 |

Cell cycle

| SW480 cell cycle | | G1 |  | S |  | G2/M |
| --- | --- | --- | --- | --- | --- | --- |
|  | CS | 40.51 |  | 23.56 |  | 35.92 |
|  | KD-59 | 52.19 |  | 33.81 |  | 14 |
|  | KD-61 | 53.89 |  | 45.47 |  | 0.64 |
|  |  |  |  |  |  |  |
|  |  | G1 |  | S |  | G2/M |
| DLD-1 cell cycle | CS | 48.93 |  | 18.52 |  | 32.55 |
|  | KD-59 | 47.7 |  | 29.31 |  | 22.99 |
|  | KD-61 | 44.5 |  | 39.63 |  | 15.87 |

Apoptosis 1

| SW480 |  | CS |  |  | KD-59 |  |  | KD-61 |  |  |
| --- | --- | --- | --- | --- | --- | --- | --- | --- | --- | --- |
|  | CDDP(-) | 10.14 | 9.02 | 9.55 | 7.83 | 8.55 | 8.17 | 8.8 | 8.53 | 8.62 |
|  | CDDP(+) | 10.23 | 9.15 | 9.63 | 11.97 | 12.58 | 12.15 | 12.5 | 13.05 | 12.95 |
|  |  |  |  |  |  |  |  |  |  |  |
|  |  |  |  |  |  |  |  |  |  |  |
|  |  | CS | | | KD-59 | | | KD-61 | | |
| DLD-1 | CDDP(-) | 6.5 | 6.46 | 6.55 | 5.85 | 5.72 | 5.96 | 7.14 | 7.21 | 7.05 |
|  | CDDP(+) | 14.69 | 14.51 | 14.76 | 19.39 | 19.51 | 19.29 | 19.99 | 21.01 | 18.98 |
|  |  |  |  |  |  |  |  |  |  |  |
|  |  |  |  |  |  |  |  |  |  |  |
|  |  | CS | | | KD-59 | | | KD-61 | | |
| SW480 | 5-FU(-) | 28.11 | 27.52 | 28.48 | 30.76 | 31.24 | 28.6 | 29.35 | 30.35 | 28.2 |
|  | 5-FU(+) | 59.1 | 58.34 | 59.56 | 69.51 | 71.02 | 67.97 | 75.25 | 76.11 | 74.24 |
|  |  |  |  |  |  |  |  |  |  |  |
|  |  |  |  |  |  |  |  |  |  |  |
|  |  | CS | | | KD-59 | | | KD-61 | | |
| DLD-1 | 5-FU(-) | 6.35 | 5.05 | 5.68 | 3.65 | 4.45 | 3.96 | 6.03 | 4.52 | 5.24 |
|  | 5-FU(+) | 30.45 | 28.09 | 29.31 | 37.14 | 40.12 | 38.45 | 45.58 | 48.07 | 46.72 |

Apoptosis 2

| SW480 |  | CS |  |  | KD-59 |  |  | KD-61 |  |  |
| --- | --- | --- | --- | --- | --- | --- | --- | --- | --- | --- |
|  | Oxaliplatin (-) | 3.57 | 5.94 | 7.21 | 8.43 | 2.49 | 4.23 | 5.12 | 8.50 | 5.18 |
|  | Oxaliplatin (+) | 12.13 | 15.28 | 9.51 | 23.85 | 29.74 | 24.17 | 30.17 | 21.84 | 17.07 |
|  |  |  |  |  |  |  |  |  |  |  |
|  |  |  |  |  |  |  |  |  |  |  |
|  |  | CS | | | KD-59 | | | KD-61 | | |
| DLD-1 | Oxaliplatin (-) | 4.62 | 8.36 | 3.09 | 7.36 | 5.81 | 2.06 | 7.99 | 5.26 | 4.08 |
|  | Oxaliplatin (+) | 15.51 | 9.58 | 12.49 | 26.93 | 32.54 | 27.38 | 34.86 | 21.98 | 24.69 |
|  |  |  |  |  |  |  |  |  |  |  |
|  |  |  |  |  |  |  |  |  |  |  |
|  |  | CS | | | KD-59 | | | KD-61 | | |
| SW480 | CPT-11(-) | 1.49 | 5.73 | 5.28 | 2.46 | 6.87 | 4.99 | 7.11 | 3.21 | 2.06 |
|  | CPT-11(+) | 12.15 | 15.25 | 11.30 | 32.52 | 39.12 | 29.77 | 28.94 | 38.65 | 37.99 |
|  |  |  |  |  |  |  |  |  |  |  |
|  |  |  |  |  |  |  |  |  |  |  |
|  |  | CS | | | KD-59 | | | KD-61 | | |
| DLD-1 | CPT-11 (-) | 2.35 | 6.18 | 7.01 | 7.60 | 8.10 | 5.16 | 8.19 | 3.01 | 6.82 |
|  | CPT-11 (+) | 11.35 | 19.57 | 18.53 | 25.07 | 35.28 | 27.05 | 33.52 | 23.37 | 25.26 |

Tumor xenograft models

| Volume | Day | Control | | | | | CDDP | | | | | Birc6 knockdown | | | | | CDDP+Birc6 knockdown | | | | |
| --- | --- | --- | --- | --- | --- | --- | --- | --- | --- | --- | --- | --- | --- | --- | --- | --- | --- | --- | --- | --- | --- |
|  | 7 | 54 | 43 | 26 | 55 | 34 | 26 | 64 | 34 | 46 | 29 | 48 | 29 | 54 | 27 | 47 | 23 | 43 | 38 | 37 | 54 |
|  | 14 | 358 | 187 | 232 | 215 | 273 | 172 | 199 | 231 | 254 | 201 | 264 | 137 | 206 | 165 | 212 | 103 | 134 | 154 | 102 | 98 |
|  | 21 | 408 | 256 | 361 | 321 | 438 | 249 | 271 | 368 | 339 | 310 | 401 | 251 | 327 | 281 | 305 | 143 | 151 | 129 | 162 | 136 |
|  | 28 | 681 | 506 | 793 | 636 | 739 | 381 | 402 | 403 | 483 | 496 | 603 | 524 | 551 | 503 | 498 | 189 | 231 | 205 | 293 | 181 |
|  |  |  |  |  |  |  |  |  |  |  |  |  |  |  |  |  |  |  |  |  |  |
| Weight(g) | Control | CDDP | Birc6 knockdown | CDDP+Birc6 knockdown | | |  |  |  |  |  |  |  |  |  |  |  |  |  |  |  |
|  | 0.632 | 0.528 | 0.602 | 0.183 |  |  |  |  |  |  |  |  |  |  |  |  |  |  |  |  |  |
|  | 0.785 | 0.575 | 0.542 | 0.089 |  |  |  |  |  |  |  |  |  |  |  |  |  |  |  |  |  |
|  | 0.529 | 0.302 | 0.493 | 0.136 |  |  |  |  |  |  |  |  |  |  |  |  |  |  |  |  |  |
|  | 0.473 | 0.393 | 0.386 | 0.102 |  |  |  |  |  |  |  |  |  |  |  |  |  |  |  |  |  |
|  | 0.497 | 0.302 | 0.342 | 0.174 |  |  |  |  |  |  |  |  |  |  |  |  |  |  |  |  |  |

Data for cell proliferation assay, colony formation assay, cell cycle and apoptosis assay and tumor xenograft models.
