# Supplementary figures and images for: Overexpression of BIRC6 Is a Predictor of Prognosis for Colorectal Cancer
Source: PLoS One. 2015 May 1;10(5):e0125281. doi: 10.1371/journal.pone.0125281 (PMC4416929; doi:10.1371/journal.pone.0125281)

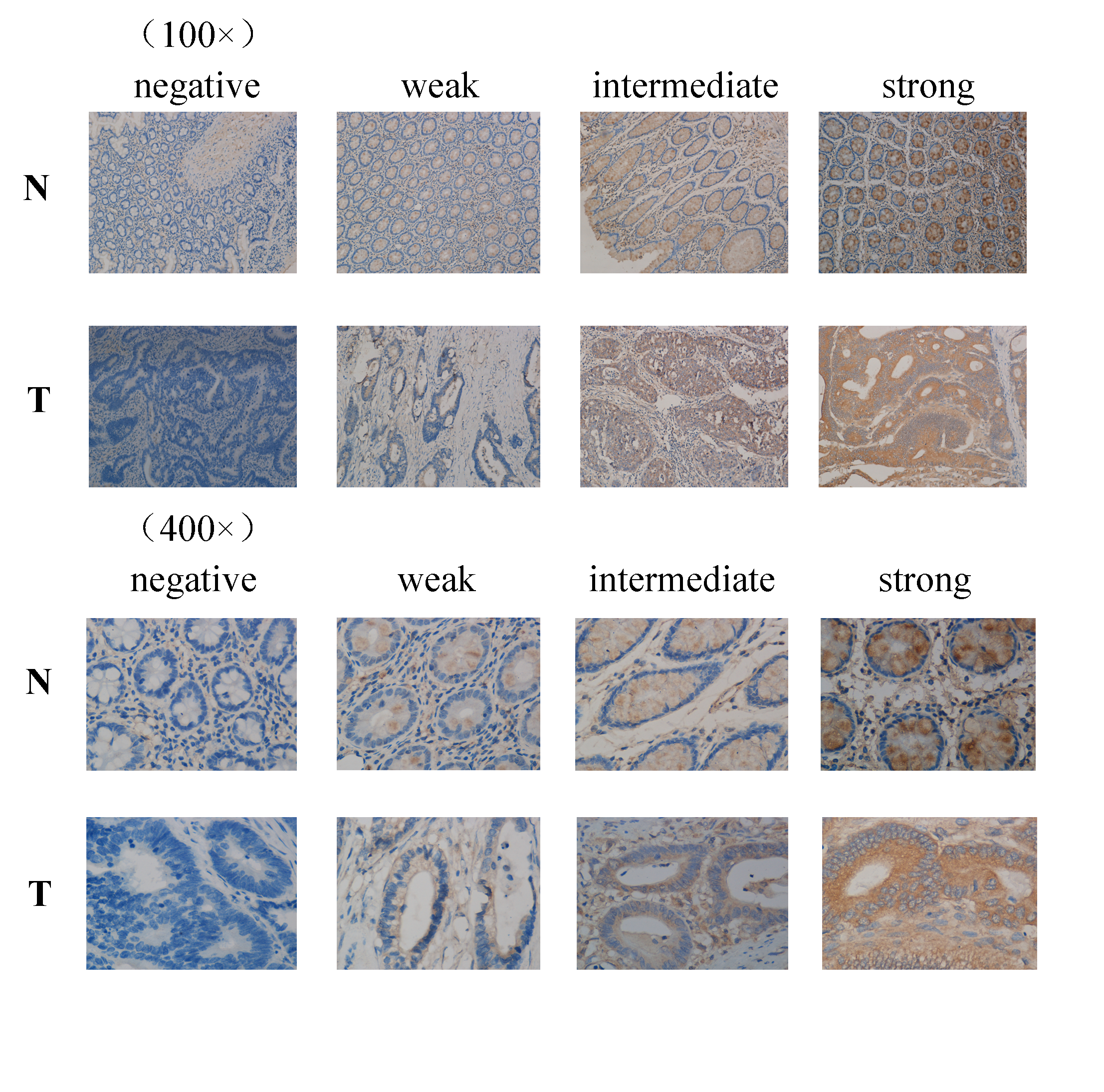

Supplement: S1 Fig — Representative images of immunohistochemistry. (TIF) [file pone.0125281.s003.tif]

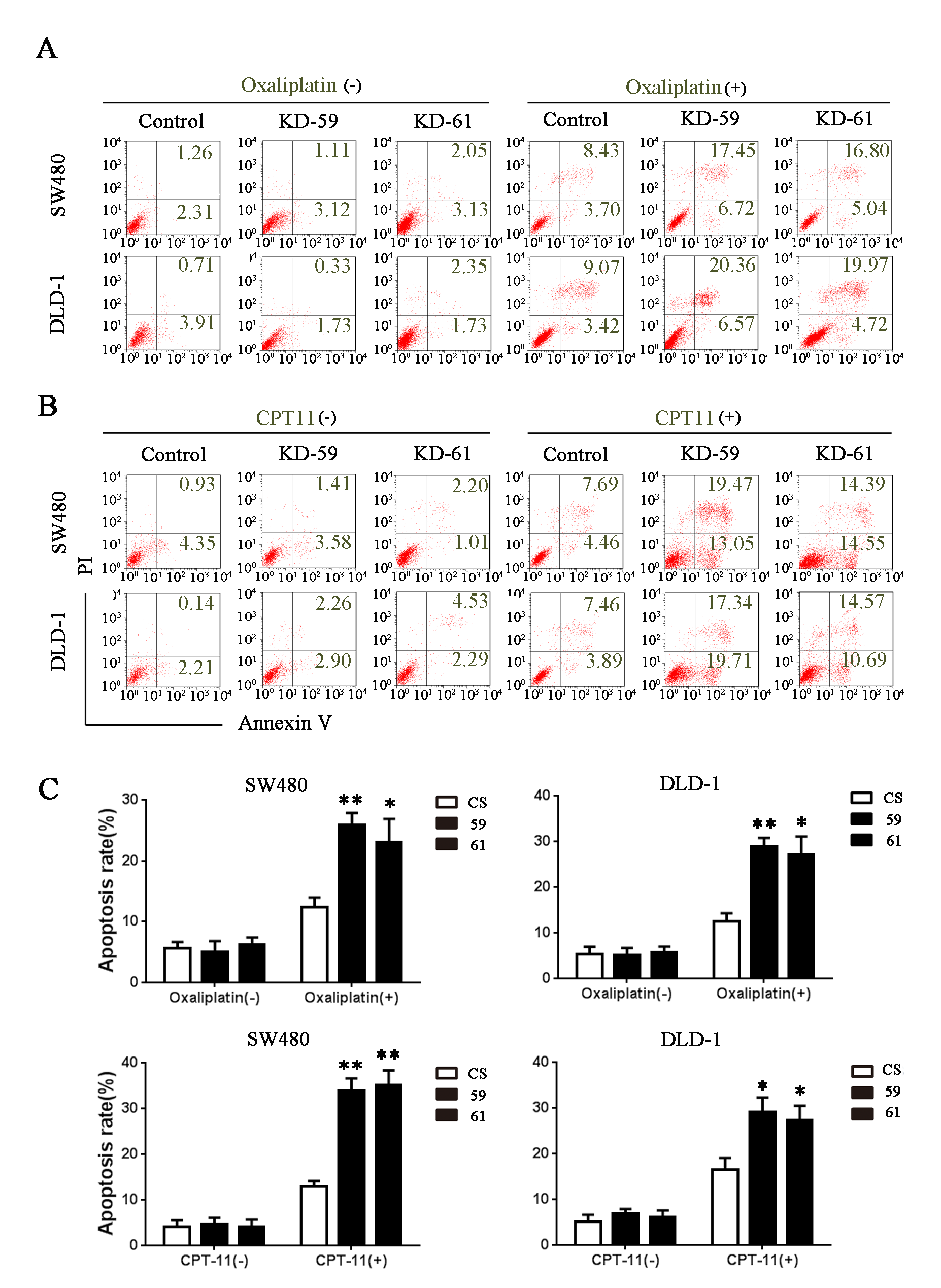

Supplement: S2 Fig — (A) Representative images of cell apoptosis assessed by Annexin V/PI staining in the control (CS) and BIRC6 knockdown cells treated with oxaliplatin (10 μM for SW480 and DLD-1) for 48 h or not. (B) Representative images of cell apoptosis for the control and BIRC6 knockdown cells treated with CPT-11 (4 μM for SW480 and DLD-1) for 24 h or not. (C) Quantification of the apoptosis rate. Data present Mean ± SEM from three independent experiments. *, P < 0.05; **, P < 0.01 vs. control. (TIF) [file pone.0125281.s004.tif]
