# Supplementary material for: Overexpression of BIRC6 Is a Predictor of Prognosis for Colorectal Cancer
Source: PLoS One. 2015 May 1;10(5):e0125281. doi: 10.1371/journal.pone.0125281 (PMC4416929; doi:10.1371/journal.pone.0125281)
Supplement: S1 Table — (DOCX) [file pone.0125281.s005.docx]

Table S1. Characteristics of 126 CRC patients.

| Characteristics |  | CRC patients |
| --- | --- | --- |
| patients |  | 126 |
| Age (years) | ≤50 | 34 (26.98%) |
|  | ＞50 | 92 (73.02%) |
| Gender | male | 71 (56.35%) |
|  | female | 55 (43.65%) |
| Tumor location | colon | 72 (57.14%) |
|  | rectal | 54 (42.86%) |
| Tumor size (cm) | ≤5cm | 107 (84.92%) |
|  | ＞5cm | 19 (15.08%) |
| Invasion depth | T1-T2 | 23 (18.25%) |
|  | T3-T4 | 103 (81.75%) |
| Lymph node metastasis | N0 | 72 (57.14%) |
|  | N1-N2 | 54 (42.86%) |
| Distant metastasis | M0 | 102 (80.95%) |
|  | M1 | 24 (19.05%) |
| TNM stage | I-II | 62 (49.21%) |
|  | III-IV | 64 (50.79%) |
| Tumor histology type | adenocarcinoma | 108 (85.71%) |
|  | mucinous/signet-ring | 18 (14.29%) |
| Tumor degree of differentiation | well/moderate | 82 (65.08%） |
|  | poor | 44（34.92%） |
| KRAS status | Wild type | 89 (70.63%） |
|  | Mutation | 37 (29.37%） |
| MSI status | MSS | 104 (82.54%） |
|  | MSI | 22 (17.46%） |
| Chemotherapy | yes | 42（33.33%） |
|  | no | 84（66.67%） |
| Expression of BIRC6 | positive | 73 (57.94%) |
|  | negative | 53 (42.06%) |

Clinical features of 126 CRC patients. Abbreviations: TNM, tumor-node-metastasis. MSI, microsatellite instability; MSS, microsatellite stable.
